# Death associated protein like 1 acts as a novel tumor suppressor in melanoma by increasing the stability of P21 protein

**DOI:** 10.1007/s11010-024-05067-0

**Published:** 2024-07-09

**Authors:** Xiaoyan Liu, Xiaojuan Hu, Meiyu Jing, Lijin Huang, Yaqi You, Yaru Zhang, Ke Li, Yunhai Tu, Youjia Liu, Xiaogang Chen, Jianzhong Su, J. Fielding Hejtmancik, Ling Hou, Xiaoyin Ma

**Affiliations:** 1 Laboratory of Developmental Cell Biology and Disease, State Key Laboratory of Ophthalmology, Optometry and Visual Science, Eye Hospital, Wenzhou Medical University, Wenzhou, 325027 China; 2 National Engineering Research Center of Ophthalmology and Optometry, Eye Hospital, Wenzhou Medical University, Wenzhou, 325027 China; 3 National Clinical Research Center for Ocular Diseases, Eye Hospital, Wenzhou Medical University, Wenzhou, 325027 China; 4 State Key Laboratory of Ophthalmology, Optometry and Visual Science, Eye Hospital, Wenzhou Medical University, Wenzhou, 325027 China; 5 Ophthalmic Genetics and Visual Function Branch, National Eye Institute, National Institutes of Health, Bethesda, MD 20892 USA

**Keywords:** Melanoma, Cell proliferation, DAPL1, P21, Protein stability

## Abstract

Melanoma is a primary malignant tumor with high lethality, which occurs in the skin and eye tissues, while the molecular mechanisms of melanomagenesis remain largely unknown. Here, we show that death-associated protein-like 1 (DAPL1) expression is lower in melanoma tissues than in paracancerous tissues or nevus tissues, and Uveal melanoma patients with lower DAPL1 expression have a poorer survival rate than those with higher expression of DAPL1. Overexpression of DAPL1 inhibits proliferation of cultured melanoma cells, whereas knockdown of DAPL1 increases cell proliferation. Tumor transplantation experiment results also demonstrate that DAPL1 inhibits tumorigenesis of melanoma cells both in subretinal and subcutaneous tissues of nude mice in vivo. Finally, DAPL1 inhibits proliferation of melanoma cells by increasing the protein level of P21 via decreasing the ubiquitin mediated degradation of P21 and promoting its stability. Conversely, knockdown of P21 neutralizes the effects of inhibition of DAPL1 on melanoma cell proliferation and enhances the severity of melanoma tumorigenesis. These results suggest that DAPL1 is a novel melanoma tumor suppressor gene and thus a potential therapeutic target for melanoma.

**Supplementary Information:**

The online version contains supplementary material available at 10.1007/s11010-024-05067-0.

## Introduction

Melanoma is a malignant tumor with high mortality and poor prognosis, which includes Cutaneous melanoma (CM) and Uveal melanoma (UM). UM is the most common primary intraocular malignancy in adults [1], while CM accounts for 75% of the mortality rate of skin malignant tumors [2]. UM and CM derive from neural crest-derived melanocytes, share the same embryonic origin, and display similar cellular functions. However, they have differences in their genetic alterations and biological behavior [3]. In CM, the mutations of BRAF (v-RAF murine sarcoma viral oncogene homolog B) or NRAS (neuroblastoma RAS viral oncogene homolog) active the mitogen-activated protein kinase (MAPK) and the phosphatidylinositol-3-kinase/AKT serine/threonine kinase 1 (PI3K/AKT) pathways were reported to play critical roles [4–7]. On the other hand, some reports showed that most UM cells carry GNAQ^Q209L^ or GNA11^Q209L^ mutation, also named GNAQ/11 [8]. Despite improvements in the understanding of the intrinsic mechanisms fostering melanoma and in developing new therapeutic strategies, the treatments needed for this disease still need to be fulfilled. A deeper understanding of the molecular mechanisms of melanoma tumorigenesis may facilitate a better understanding of melanoma pathogenesis and provide an actionable therapeutic strategy to improve treatment outcomes.

Death-associated protein-like 1 (DAPL1, also known as early epithelial differentiation-associated protein, EEDA) was first identified in skin epithelial cells [1], while its biological functions are largely unknown. In the zebrafish Daplb (the zebrafish ortholog of DAPL1 in mammals) acts as a translational inhibitor in zebrafish oocytes by complexing with eIF5a and then stably inserting into the ribosome polypeptide exit tunnel. Addition of recombinant zebrafish Dap1b protein to a mammalian translation extract in vitro blocks translation and reconstitutes the dormant ribosome state [2]. DAPL1 is also a testosterone production regulator in Leydig cells of the mouse testis [3]. Knockdown of DAPL1 expression in fetal pituitary cells suppresses expression of growth hormone and cyclin D2, suggesting that DAPL1 might be involved in regulating cell proliferation and development [4].

DAPL1 is a candidates for hepatoblastoma tumorigenesis [5]. Hypomethylation of DAPL1 also is associated with the prognosis of the EGFR Del19 mutation subtype in lung cancer patients, and lung cancer patients showing hypomethylation of DAPL1 have significantly longer overall survival times [6]. Finally, DAPL1 is a crucial regulator of CD8 T cell immunity and a potential target for cancer immunotherapy [7].

DAPL1 is a crucial regulator of retinal pigment epithelial cell proliferation, epithelial-mesenchymal transition (EMT), and antioxidant defense systems [7–10]. Moreover, we also revealed that DAPL1 exists in a positive feedback loop with microphthalmia-associated transcription factor (MITF) [9, 10], which is a critical regulator of melanocyte development [11–13] and acts as a tumor suppressor in melanoma [12, 14–16]. However, the biological function of DAPL1 in melanoma and the underlying mechanisms remain unclear.

In the present study, we show that DAPL1 expression in melanoma is low relative to that in paracancerous tissues and nevus tissues. Survival analysis suggests that patients with low expression of DAPL1 have poorer overall survival rates than those with high DAPL1 expression. Overexpression of DAPL1 in melanoma cells inhibits cell proliferation in vitro and tumor growth in vivo. Conversely, knockdown of DAPL1 in melanoma cells increases cell proliferation. Mechanistically, DAPL1 inhibits ubiquitination mediated degradation of P21 and promotes its stability, inhibiting the proliferation of melanoma cells. Knockdown of P21 neutralizes the inhibition of melanoma cell proliferation by DAPL1 in vitro and enhances the severity of melanoma tumorigenesis in nude mice in vivo. These results suggest that DAPL1 is a novel melanoma tumor suppressor and a potential therapeutic target for melanoma.

## Materials and methods

### Melanoma tissue specimens

A total of 3 paired specimens of tumor and adjacent non-tumor tissues were collected from 3 UM patients. All research involving the clinical samples was approved by the ethics committee of Wenzhou Medical University Eye Hospital (2022-043-K-28-02) and patients gave informed consent consistent with the tenets of the Declaration of Helsinki.

### Animals

BALB/C male nude mice were purchased from Zhejiang Vitalihua Laboratory Animal Technology Co and were maintained in the pathogen-free facility of Wenzhou Medical University. Ethical approval was granted by the ethics committee of Wenzhou Medical University (× 2022-3004).

### In vivo tumor growth assay

The 6-week-old male nude mice were randomly divided into two groups. For xenograft tumorigenicity assays 2 × 10^6^ tumor cells suspended in phosphate-buffered saline were injected subcutaneously into the flanks of the nude mice. For ocular orthotopic tumor experiments, a 2 μl cell suspension containing 1.3 × 10^5^ UM cells was injected subretinally under microscope guidance in the right eye of each mouse, and postoperative care was performed. The mice were euthanized one month later after the xenotransplantation. All eyeballs or the subcutaneous UM tumors were isolated and fixed for further analysis. The length and width of the tumors were measured with a Vernier caliper after sacrifice, and the tumor volume was calculated according to the formula V = L × W^2^ × 0.5.

### Cell culture

A375 cells, MuM-2C cells & C918 cells were purchased from the National Collection of Authenticated Cell Cultures and Qingqi (Shanghai) Biotechnology Development Co., Ltd., respectively. The cells were cultured in complete Dulbecco’s modified Eagle’s medium (DMEM; Gibco, #10,938) supplemented with 10% (vol: vol) fetal bovine serum (FBS; Gibco, #16,140,071) and 1% (vol: vol) antibiotics (penicillin/streptomycin; Beyotime, # C0222) in a humidified incubator with 5% CO2 at 37 °C.

### RNA extraction and real-time PCR

Total RNA was extracted using TRIzol (Invitrogen, # 15,596–018) according to the manufacturer’s instructions. cDNA was synthesized using the PrimeScript RT reagent kit (Promega, # c1181) and the SYBR Green Quantitative kit (Applied Biosystems, #4,367,659) with the following procedures: 50 °C for 2 min, 95 °C for 10 min, 40 cycles of 95 °C for 15 s and 60 °C for 1 min. The changes in the mRNA levels were quantified using GAPDH mRNA as the control. The primers used in this study were as follows: GAPDH-F: AGGTCGGTGAACGGATTTG, GAPDH-R: TGTAGACCATGTAGTTGAGGTCA; DAPL1-F: GAAAGCTGGAGGGATGCGAA; DAPL1-R: TGATGTCCGTGTGAACTGT; P21-F: AGTCAGTTCCTTGTGGAGCC; P21-R: CATTAGCGCATCACAGTCGC.

### Western blot

Melanoma cells were washed with phosphate-buffered saline (Beyotime, # C0221A) and lysed on ice for 30 min with radioimmunoprecipitation assay (Beyotime, # P0013B). Twenty micrograms of protein lysate was separated by 10% SDS-PAGE and transferred to polyvinylidene fluoride membranes. The membranes were then blocked with 5% skimmed milk at room temperature for 2 h and incubated at 4 °C overnight with anti-DAPL1 (1:1,000, Abcam, # ab150969), anti-P21 (1: 1,000, CST, # 2947S), anti-E2F1 (1: 1,000, Abcam, # ab179445), anti-IgG (1: 1,000, Sigma-Aldrich, # PP64), anti-Ubiquitin (1: 1,000, Abcam, # ab134953), or anti-GAPDH (1: 1000, aksomics, # KC-5G4). The membranes were then probed with fluorescein-conjugated secondary antibodies (1:5000, LI-COR, # 926-32,213, # 926-32,212) at room temperature for 2 h. Finally, the blots were analyzed using the Odyssey CLx system (LI-COR, USA), and quantitative densitometry of the bands was performed using ImageJ software. Cycloheximide (# HY12320) and MG-132 (#HY-13259) were purchased from MedChemExpress Corporation.

### Cell viability assay

For the CCK-8 cell growth assay, melanoma cells were seeded at a density of 1000 cells per well in flat-bottomed 96-well plates, and at the end of the incubation time, 10 μl of Cell Counting Kit-8 (CCK-8; Beyotime, # C0043) was added to each well. After 2 h, the optical density at 450 nm was determined using a microplate reader (Molecular Devices, USA). For the colony formation assay, 2000 cells/well for melanoma cells were seeded into six-well plates. After 10 days, the plates were stained with 1% crystal violet (Sangon Biotech, # E607309) and photographed.

### EdU incorporation assays

Melanoma cells were cultured in 6-well plates with 10 mmol/L EdU working solution (Beyotime, # C0078S) for 2 h at 37 °C. The cells were then fixed in 4% polyoxymethylene for 15 min and incubated with 0.3% Triton X-100 for 15 min, according to the manufacturer’s instructions. The cells were observed under a Zeiss inverted fluorescence microscope.

### Immunofluorescence assay

The cells were placed on coverslips, fixed with cold methanol, blocked with 10% fetal bovine serum for 1 h at room temperature, incubated with P21 primary antibody (1:300) at 4 °C overnight, and washed. Alexa Fluor 594 (1:200, Invitrogen, # A21207) was used to visualize the bound primary antibody. Cell micrographs were obtained using a Zeiss LSM880 confocal microscope with fixed parameters between the experimental and control group using Image J. The images were color-split, the particle edges were smoothed, and each image intensity threshold was automatically adjusted and applied.

### Gene overexpression and knockdown

DAPL1-overexpressing cells (A375 + DAPL1, C918 + DAPL1, MuM-2C + DAPL1) or EGFP-overexpressing cells (A375 + EGFP, C918 + EGFP, MuM-2C + EGFP) were produced as previously described [17]. SiRNA or shRNA were used to knockdown respective target genes. Lentivirus harboring shP21 (shRNA: aaGACCATGTGGACCTGTCAC; 10^8^ genome copies/ml) was purchased from Genechem Co. Ltd (Shanghai, China). Specific siRNAs for human DAPL1 were synthesized by Gene Pharma Co (Shanghai, China) with the following sequences: si-DAPL1-1, GCACUUGCUUGGUAAAUUATT; si-DAPL1-2, GCUGGAGGAAUGAGAAUUUTT; si-NC, AUUUCUUUCAUGUUGUGGGTT. For transfections, 4 μl of siRNA and 4 μl of Lipofectamine™ 2000 (SignaGen, # SL100468) were diluted with 200 μl of buffer medium and mixed after 10 min incubation. Then added dropwise to each culture well containing 800 μl of serum-free DMEM medium (final siRNA concentration, 100 nM). The medium was discarded 6 h thereafter and replaced with fresh complete culture medium with 10% FBS. Experiments such as western blotting were performed after culturing for 48 h.

### Statistical analysis

Kaplan–Meier survival curves were used to estimate differences in patient outcomes and statistical differences in survival were estimated using the Wilcoxon rank-sum test and the Cox proportional hazards regression. Each cellular experiment was repeated three times, while each mouse experiment was carried out using 6 mice. All quantitative data were presented as the mean ± SEM. The statistical significance of differences between groups was obtained using one-way ANOVA in GraphPad (San Diego, CA). Correlations in expression levels were estimated using linear regression. Differences were considered to be significant at P < 0.05.

## Results

### DAPL1 is lowly expressed in melanoma tissues and confers an unfavorable prognosis

**Fig. 1:**
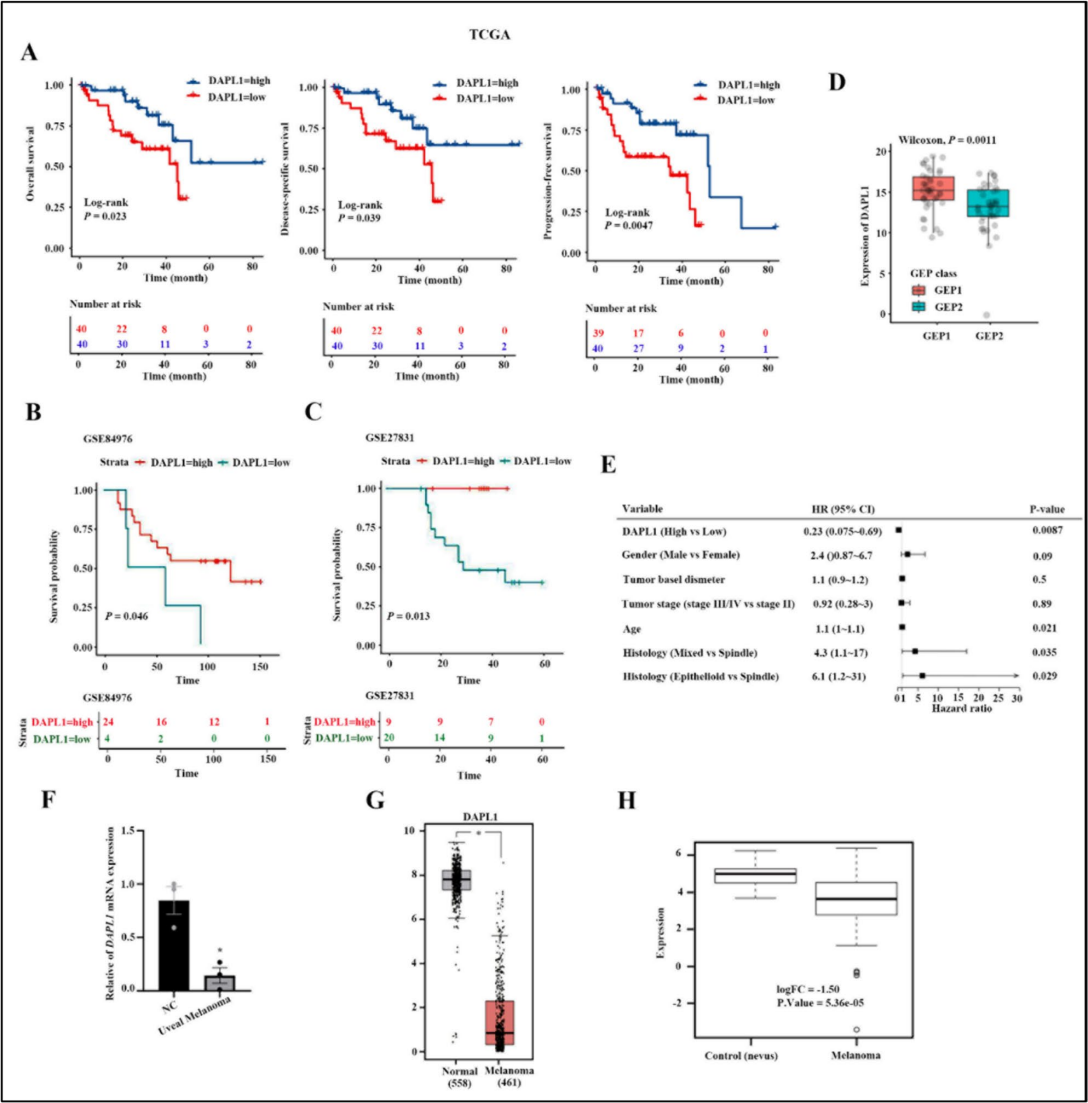
DAPL1 is lowly-expressed in melanoma tissues. **A** Kaplan–Meier curves were calculated for OS, DSS, and PFS in patients with DAPL1 high and low expression in the TCGA-UM cohort. **B**, **C** Kaplan–Meier overall survival curves of the two UM groups with DAPL1 high and low expression in the GEO-UM cohort. **D** Boxplots of the differences of DAPL1 between GEP1 and GEP2 UM patients. The Wilcoxon rank-sum test determined statistical differences. **E** Forest plot of the multivariate Cox proportional hazards regression model. **F** Real-time PCR analysis of *DAPL1* mRNA expression in uveal melanoma and adjacent tissues. **G** The expression level of DAPL1 was analyzed in the skin cutaneous melanoma and normal skin tissues based on the data from TCGA database. **H** The expression level of DAPL1 was analyzed in the skin cutaneous melanoma and the nevus tissues based on the data from GSE98394. Values are mean ± SE (n ≥ 3); * indicates P < 0.05. DSS, disease-specific survival; GEO, Gene Expression Omnibus database. GEP, gene expression profile; OS, overall survival; PFS, progression-free survival; TGCA, The Cancer Genome Atlas

To evaluate the association between DAPL1 and the prognosis of UM patients, we performed survival analysis in the TCGA-UM cohort, comparing patients with high and low DAPL1 expression based on their median values. The overall survival (OS), disease-specific survival (DSS), and progression-free survival (PFS) were analyzed and compared between the DAPL1-low expression and DAPL1-high expression groups (Fig. 1A). Low expression of DAPL1 was significantly associated with poorer OS, DSS, and PFS in UM patients (log-rank P < 0.05 for all). Consistent with this, in the GEO-UM cohort, Kaplan–Meier curves also showed that patients with a low expression of DAPL1 were significantly associated with poorer OS (log-rank P < 0.05) (Fig. 1B, C). The prognostic 12-gene expression profile (12-GEP), which stratifies UM tumors into three molecular subtypes corresponding to either low- (Class 1A), intermediate- (Class 1B), or high-risk (Class 2) of metastasis [18, 19] test is commonly used to guide the systemic monitoring of metastatic risk in UM patients. Expression of DAPL1 is lower in GEP2 UM patients compared with GEP1 UM patients (Fig. 1D), indicating that levels of DAPL1 expression are associated with the prognosis of UM. Multivariate Cox analysis showed that DAPL1 maintained significant associations with OS (*HR*: 0.23, 95% *CI*: 0.075–0.69, log-rank *P*: 0.0087) after adjusting for clinical variables (such as sex, age, and stage) and histologic features (Fig. 1E), consistent with DAPL1 acting as an independent prognostic factor for favorable clinical outcome.

Real-time PCR analysis of *DAPL1* mRNA expression of in UM and paracancerous tissues shows that DAPL1 expression in UM tissues is decreased relative to that in the tumor-adjacent normal tissues (Fig. 1F). Consistent with this result, we analyzed the expression of DAPL1 in the TCGA-skin melanoma cohort and compared it with the normal skin tissues. As the data shown in Fig. 1G, the DAPL1 expression level is lower in the skin melanoma tissues than in the skin tissues. Consistent with this, based on the data from GSE98394, the expression level of melanoma is also lower in the melanoma tissues than the normal nevus tissues (Fig. 1H). While these results taken together indicate that DAPL1 expression is decreased both in the CM and UM, and that this decreased DAPL1 expression is associated with unfavorable prognosis in UM patients, the biological mechanisms through which this occurs need to be delineated.

### DAPL1 inhibits melanoma cell proliferation in vitro.

**Fig. 2:**
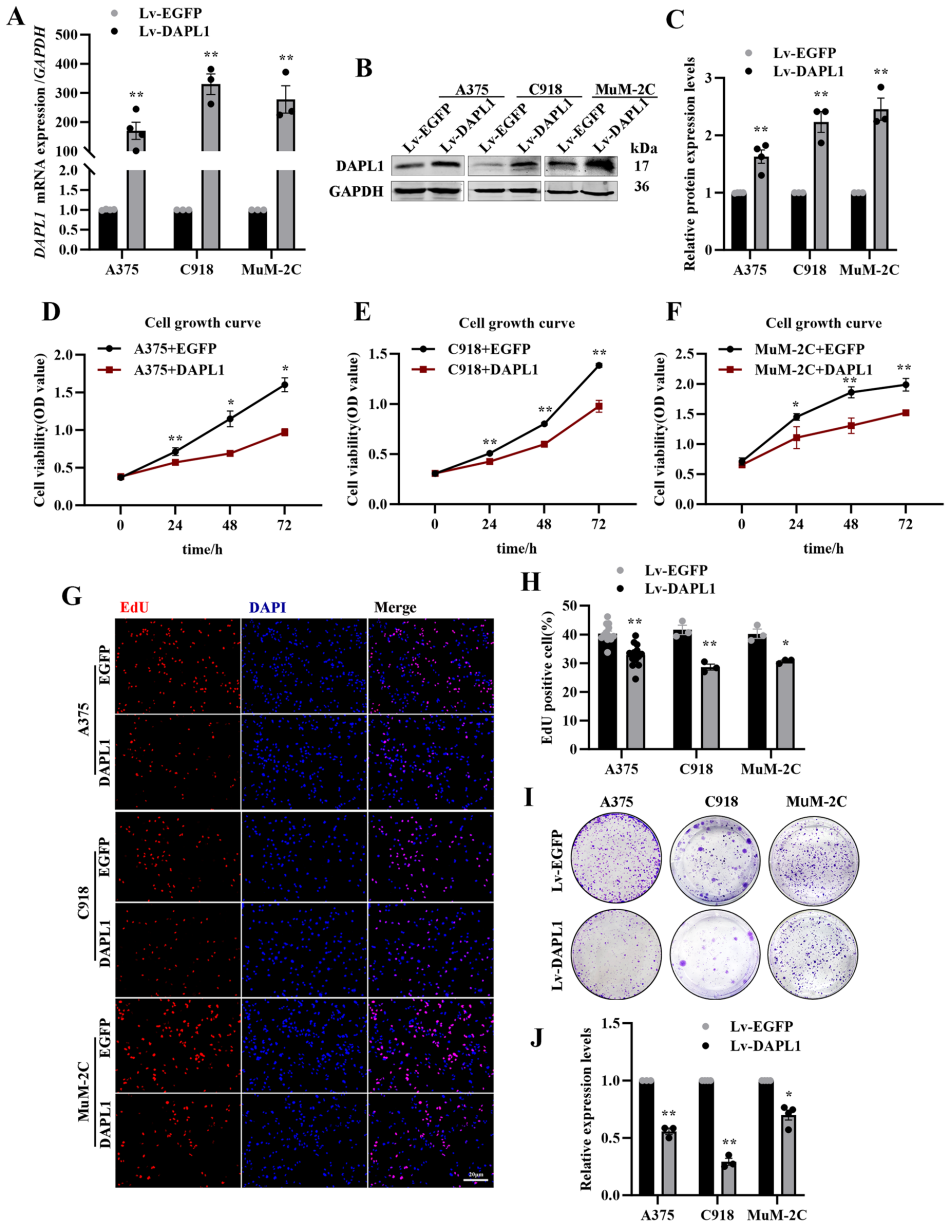
Overexpression of DAPL1 inhibits melanoma cell proliferation in vitro. **A** Real-time PCR analyzed the mRNA level of *DAPL1* in the DAPL1 or EGFP overexpressed A375, C918 and MuM-2C cells. **B**, **C** Western blot analyzed the protein level of DAPL1 in the DAPL1 or EGFP overexpressed A375, C918 and MuM-2C cells and its quantification. **D**–**F** Cell growth curve analysis of the A375, C918 and MuM2C cells with DAPL1- or EGFP- overexpression. **G**, **H** The EdU staining images and quantification of the A375, C918 and MuM2C cells with DAPL1- or EGFP- overexpression. **I**, **J** Colony formation assay images and quantification of the A375, C918 and MuM2C cells with DAPL1- or EGFP- overexpression. Scale bar: 20 μm. The data are shown as the mean ± SE of three independent experiments. N = 3. * or ** indicates p < 0.05 or p < 0.01

To evaluate the role of DAPL1 in melanoma cells, we overexpressed DAPL1 in A375, C918 and MuM-2C melanoma cell lines by lentivirus infection. As seen in Fig. 2, both mRNA and protein levels of DAPL1 were significantly increased in A375, C918 and MuM-2C cells after infection with DAPL1 lentivirus (Lv-DAPL1) (Fig. 2A, B, C). Overexpression of DAPL1 slowed down the proliferation rate of A375, C918 and MuM-2C cells (Fig. 2D–F). Similarly, overexpression of DAPL1 decreased colony formation of both A375, C918 and MuM-2C cells (Fig. 2G, H) in a colony formation assay, and A375, C918 and MuM-2C cells overexpressing DAPL1 showed lower EdU positivity than EGFP overexpression control cells (Fig. 2I, J). Taken together, all these results suggest that DAPL1 inhibits the proliferation of melanoma cells.

**Fig. 3:**
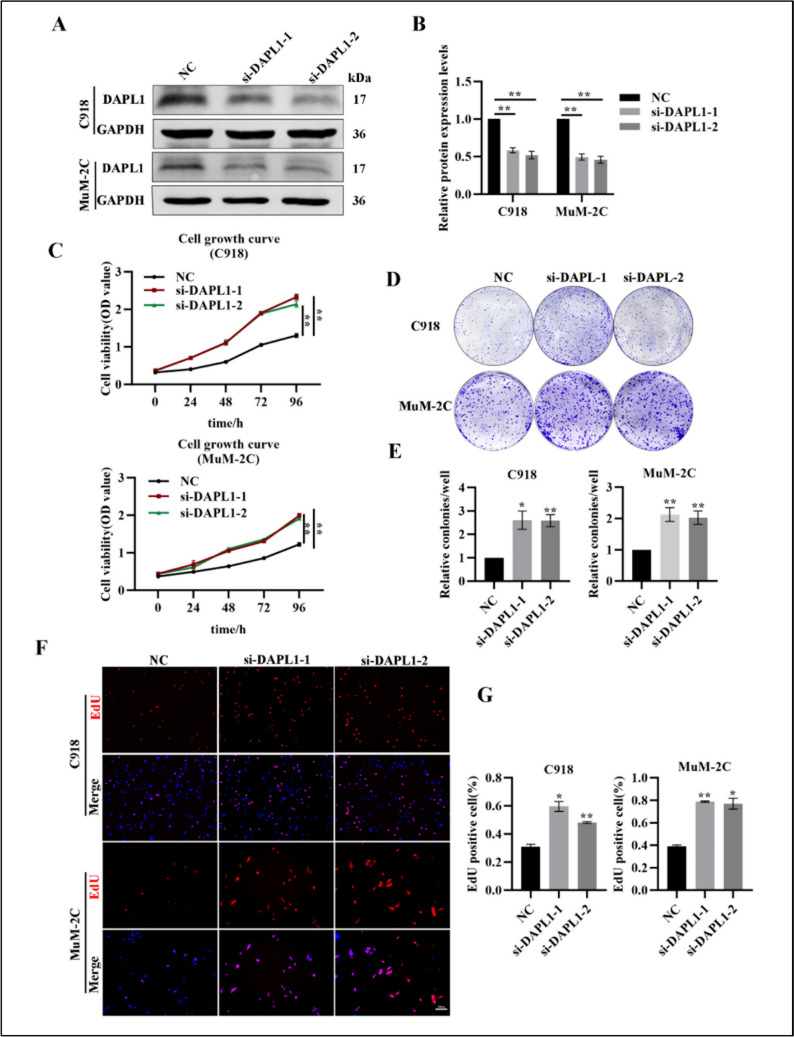
Knockdown of DAPL1 increases the proliferation of melanoma cells. **A**, **B** Western blot analyzed the protein level of DAPL1 in C918 and MuM-2C cells following the transfection of si-NC and si-DAPL1s and its quantification. **C** Cell growth curve analysis of the C918 and MuM2C cells with whether DAPL1 knockdown. **D**, **E** Colony formation assay images and quantification of the C918 and MUM2C cells with DAPL1 knockdown or not. **F**, **G** The EdU staining images and quantification of the C918 and MUM2C cells whether DAPL1 knockdown or not. The results are represented as the means ± SE of three independent experiments. N = 3. ** indicates p < 0.01. Scale bar: 20 μm

We also performed examined the effects of loss of DAPL1 function on the proliferation of melanoma cells through siRNA-mediated DAPL1 knockdown. Treatment with both si-DAPL1-1 and si-DAPL1-2 significantly decreases levels of DAPL1 protein in C918 and MuM-2C cells (Fig. 3A, B). As shown by cell growth curves, knockdown of DAPL1 significantly increases proliferation of both C918 and MuM-2C cells (Fig. 3C). Consistent with this, knockdown of DAPL1 increased colony formation in C918 and MuM-2C cells (Fig. 3 D, E). In addition, compared with the si-NC group cells, DAPL1 knockdown of DAPL1 in C918 and MuM-2C cells resulted in higher EdU-positive rates (Fig. 3 F, G). Thus, overexpression of DAPL1 inhibits melanoma cell proliferation, while knockdown of DAPL1 increases melanoma cell proliferation in cultured melanoma cells.

### DAPL1 inhibits melanoma tumor progression in vivo

**Fig. 4:**
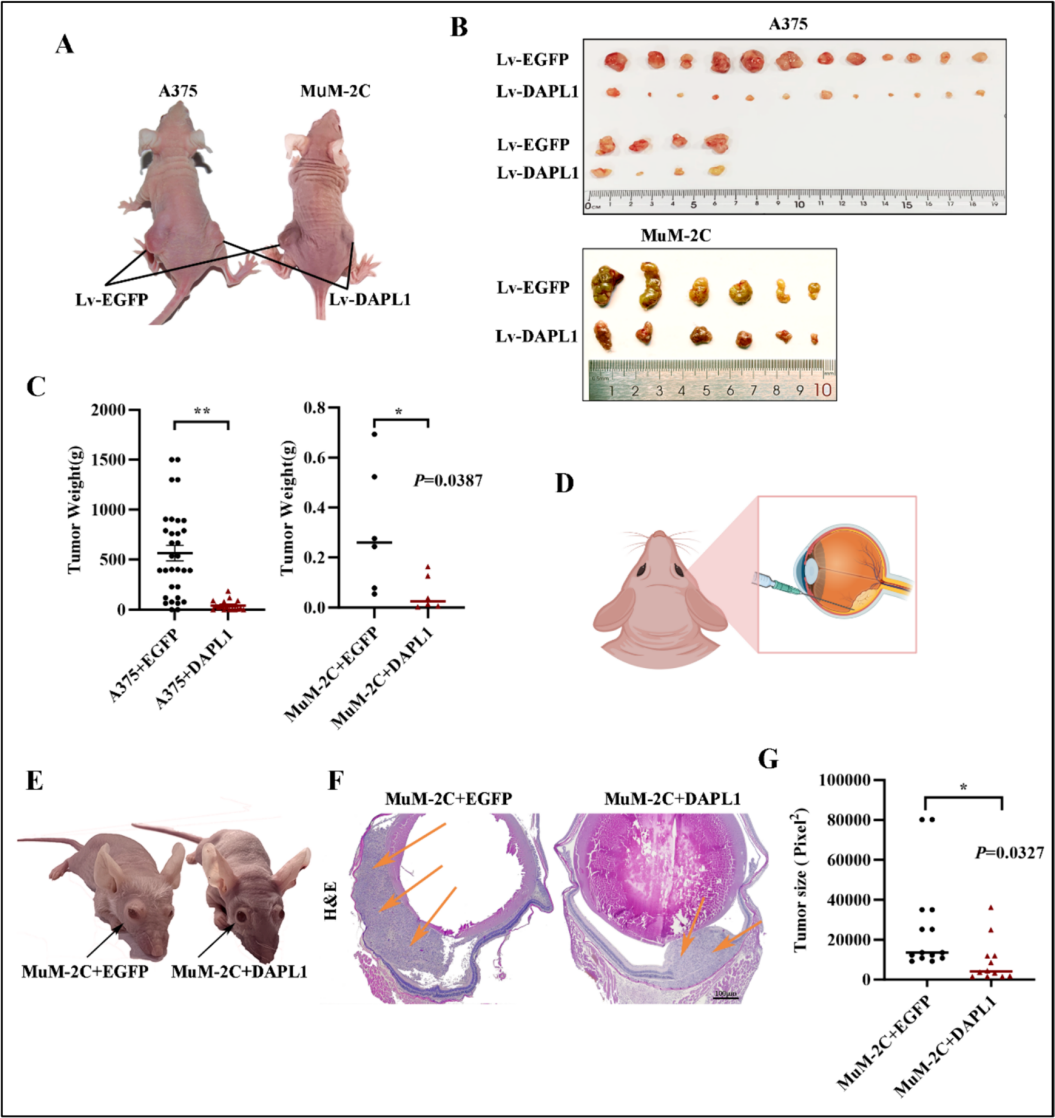
DAPL1 inhibits melanoma tumor progression in vivo. **A** A375 and MuM-2C cells with EGFP- or DAPL1-overexpressing were injected subcutaneously into nude male mice (n ≥ 6). **B**, **C** The melanoma tumors were isolated and photographed one month after the subcutaneous injection of DAPL1- or EGFP-overexpressing melanoma cells. **D** Schematic diagram of the EGFP- or DAPL1-overexpressing MuM-2C cells were subretinal injected into the right eye of male nude mice. **E** One month after the MuM-2C cells injection, the bulging, and protruding eyeballs could be observed in the EGFP- MuM-2C cells injected mice (black arrow), while the DAPL1- MuM-2C cells injected group eyes were much normal. **F**, **G** H&E staining of the eye sections after the melanoma tumor formation. The ocular melanoma size of each group was quantified with ImageJ software. Scale bar: 100 μm. The results are represented as the means ± SE of three independent experiments. * indicates p < 0.05

The above results demonstrate that DAPL1 inhibits melanoma cell proliferation in vitro, but do not address whether DAPL1 inhibits melanoma tumor growth in vivo. To clarify this, we further investigated the effect of DAPL1 on melanoma tumorigenesis in 6-week-old BALB/c male nude mice. We injected DAPL1 overexpressing A375 or MUM-2C cells into the subcutaneous of the 6-week-old BALB/c male nude mice, while equal EGFP overexpressing A375 or MUM-2C cells were used as experimental control, and analyzed tumor progression one month later. As the data shows, subcutaneous xenograft tumors developed from DAPL1 overexpressing A375 or MUM-2C cells were also significantly smaller (p < 0.05) than tumors derived from EGFP overexpressing control cells (Fig. 4A–C). Moreover, we also analyzed the tumorigenesis of DAPL1- MuM-2C and EGFP- MuM-2C cells in the subretinal of the left eye of nude mice (Fig. 4D). As the data shown, the EGFP- MuM-2C cells injected mice displayed bulging and protruding eyeballs, while the DAPL1- MuM-2C cells injected eyes appeared much more normal (Fig. 4E), suggesting that the orthotopic tumors with DAPL1-overexpressing MuM-2C cells grew more slowly than those with the EGFP-overexpressing controls. Pathological analysis of eye tissue sections showed that the average tumor size of the DAPL1-overexpressing melanoma cells was significantly smaller than the EGFP control UM cells (Fig. 4F, G). These results indicate that DAPL1 inhibits melanoma tumor growth in vivo, although the molecular mechanism through which DAPL1 inhibits UM cell proliferation is unclear.

### DAPL1 increases P21 protein levels in melanoma cells and promotes its nuclear localization.

**Fig. 5:**
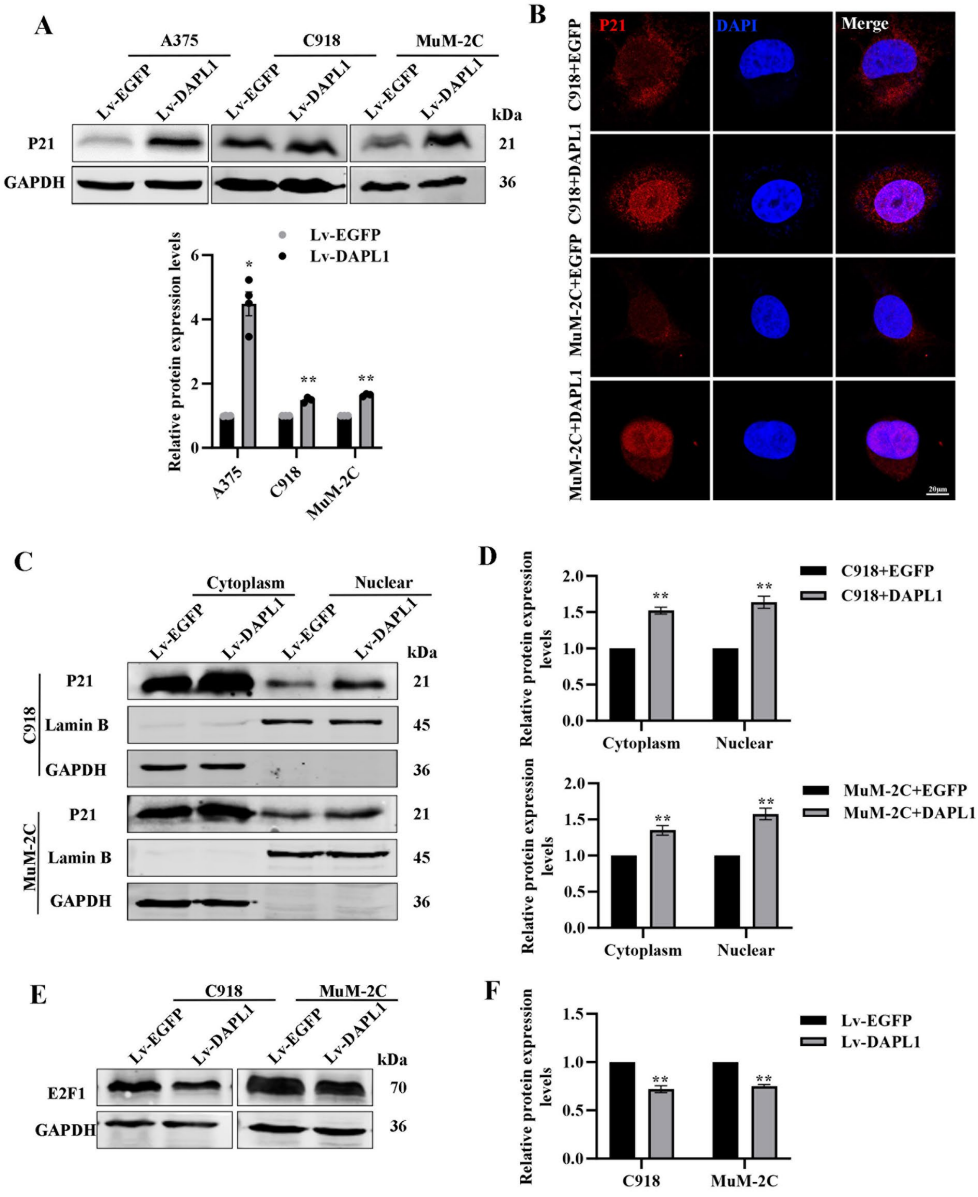
DAPL1 increases the protein level of P21 and promotes its nuclear localization in melanoma cells. **A** Western blot analyzed the protein level of P21 in DAPL1-overexpressing A375, C918 and MuM-2C cells and its quantification. **B** Immunofluorescence was used to show the P21 protein in the C918 and MuM-2C cells with EGFP- or DAPL1-overexpression. Scale bar: 20 μm. **C**, **D** Western blot analyzed the protein levels of P21 in the cytoplasmic or nuclear of C918 and MuM-2C cells with EGFP- or DAPL1-overexpression and its quantification. **E**, **F** Western blot analyzed the protein level of E2F1 in C918 and MuM-2C cells with EGFP- or DAPL1-overexpression. The means ± SE represent three independent experiment results. N = 3. ** indicates p < 0.01

The role of P21 as an oncogenic protein or tumor suppressor and its dependence on nuclear or cytoplasmic localization is well known [19, 20]. Previous work has demonstrated that DAPL1 upregulates P21 levels in RPE cells [8, 17]. In contrast, a recent study also showed that HOXA11-AS interacts with enhancers of zeste homolog 2 (EZH2) to suppress P21 expression and thus regulate cell proliferation in UM [21]. These facts prompted us to investigate whether DAPL1 also regulates P21 levels in UM cells. As shown in Fig. 5, P21 protein levels were increased in DAPL1-A375, -C918 and -MuM-2C cells as monitored by both western blot analysis and immunostaining (Fig. 5A, B).

Interestingly, we found that DAPL1 also alters the subcellular localization of P21, promoting its translocation into the nucleus as shown in the immunostaining images (Fig. 5B). Quantitating DAPL1 in the cytoplasm and nucleus indicated that DAPL1 actually increases P21 levels in the cytoplasm as well as the nucleus of both C918 and MuM-2C cells (Fig. 5C, D). Previous reports have shown that P21 regulated E2F1 in tumors [22], and our results also showed that overexpression of DAPL1 decreased E2F1 protein levels in UM cells (Fig. 5E, F), suggesting that DAPL1 not only promotes the protein levels of P21 but that this also increases its biological functions in UM cells.

### DAPL1 inhibits ubiquitin mediated degradation of P21 and promotes its stability

**Fig. 6:**
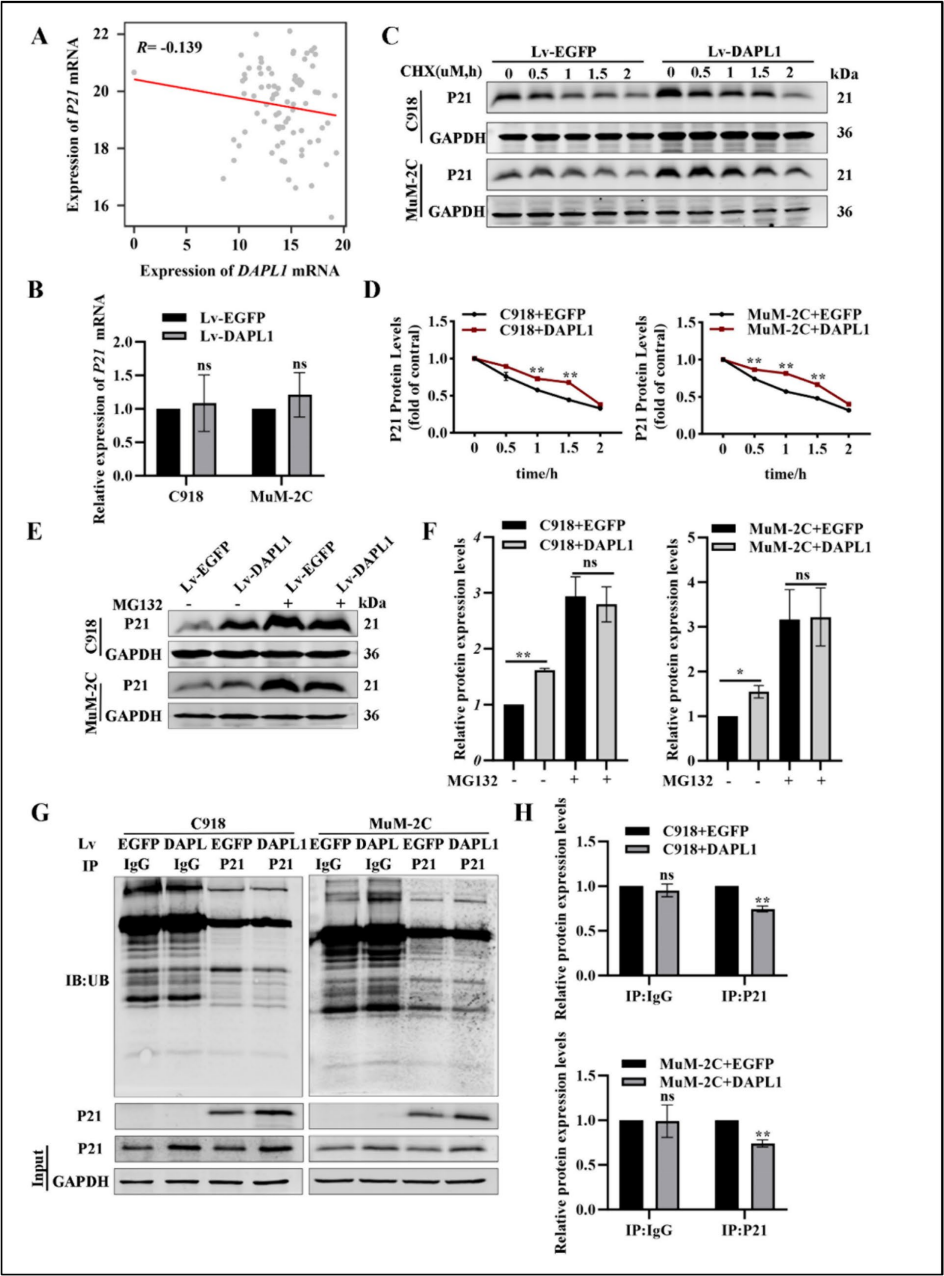
DAPL1 inhibits the ubiquitination degradation of P21 protein and promotes its protein stability. **A** Association analysis of the RNA levels of *DAPL1* and *P21* in UM tissues from the TCGA database. **B** Real-time PCR analyzed the mRNA level of *P21* in C918 and MuM-2C cells with EGFP- or DAPL1-overexpression. **C**, **D** EGFP or DAPL1 overexpressed C918 and MuM-2C cells were treated with CHX (10 μM), collected at the indicated time points, and the protein level of P21 was analyzed by western blot and its quantification. **E**, **F** EGFP or DAPL1 overexpressed C918 and MuM-2C cells were treated with MG132 (20 μg/ml) for 4 h, total protein was extracted, and the protein level of P21 was analyzed by western blot and its quantification. **G**, **H** EGFP or DAPL1 overexpressed C918 and MuM-2C cells were lysed with IP lysis/wash buffer with protease inhibitor and phosphatase inhibitor. Ubiquitin was immunoprecipitated with an anti-IgG antibody and anti-P21 antibody, and the immune precipitates were probed with anti-ubiquitin, anti-P21, and anti-GAPDH antibodies. Quantification of the ubiquitination levels of P21 relative to GAPDH expression is shown. N = 3. * indicates p < 0.05, ** indicates p < 0.01

The above results demonstrated that DAPL1 upregulates the protein level of P21 in UM cells, but the mechanism through which DAPL1 regulates P21 remained to be determined. To address this question, we analyzed mRNA levels of *P21* and *DAPL1* in the TCGA-UM cohort, which displayed no significant correlation of the mRNA levels (Fig. 6A). To confirm this result, we also analyzed the mRNA levels of *P21* in melanoma cells overexpressing DAPL1. Real-time PCR results show that overexpression of DAPL1 in C918 and MuM-2C cells did not significantly affect the mRNA level of *P21* (Fig. 6B). Next, we investigated the effect of DAPL1 levels on P21 protein level, which might occur through post-transcriptional regulation such as by modulating the stability of P21 protein. To address this question, we treated DAPL1 or EGFP overexpressed C918 and MuM-2C cells with a translation inhibitor, cycloheximide (CHX), for 0.5 to 2 h. As expected, the P21 protein had a significantly extended half-life and was relatively abundant in the C918 + DAPL1 and MuM-2C + DAPL1 cells compared with the EGFP group cells as experimental control (Fig. 6C, D). These results indicated that DAPL1 might mediate the degradation of P21 protein in UM cells, based on the results that DAPL1- and EGFP- overexpressed C918 and MuM-2C cells express similar protein levels of P21 after the treatment with proteasome inhibitor MG132 for four hours (Fig. 6E, F). Ubiquitination of P21 regulates its stability through the proteasome degradation pathway [23], and overexpression of DAPL1 reduces ubiquitination of P21 in C918 and MuM-2C cells (Fig. 6G, H). These results indicate that DAPL1 promotes P21 protein stability at least partially through modulation of the ubiquitination-mediated proteasome-dependent degradation pathway.

### Knockdown of P21 suppresses DAPL1-dependent inhibition of melanoma tumor growth

**Fig. 7:**
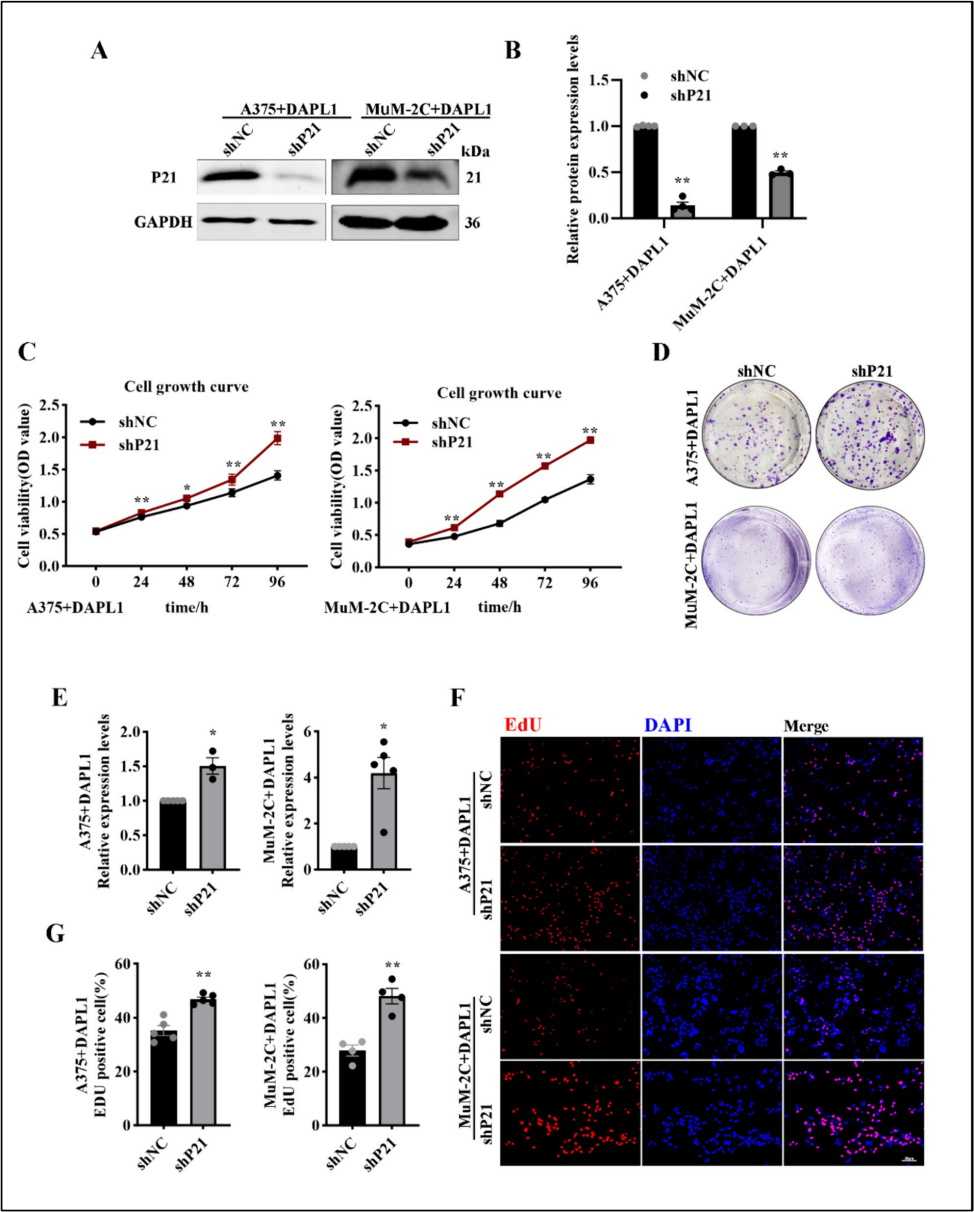
Knockdown of P21 suppresses DAPL1-dependent inhibition of melanoma cell proliferation in vitro. **A**, **B** Western blot analyzed the protein level of P21 in A375 + DAPL1 and MuM-2C + DAPL1 cells after lentiviral shP21 mediated P21 knockdown and its quantification. **C** Cell growth curve analysis of the A375 + DAPL1 and MuM2C + DAPL1 cells with shP21 mediated P21 knockdown or not. **D**, **E** Colony formation assay images and quantification of the A375 + DAPL1 and MuM-2C + DAPL1 cells with shP21 mediated P21 knockdown or not. **F**, **G** The EdU staining images and quantification of the A375 + DAPL1 and MuM-2C + DAPL1 cells with shP21 mediated P21 knockdown or not. The means ± SE represent three independent experimental results. N = 3. ** indicates p < 0.01

**Fig. 8:**
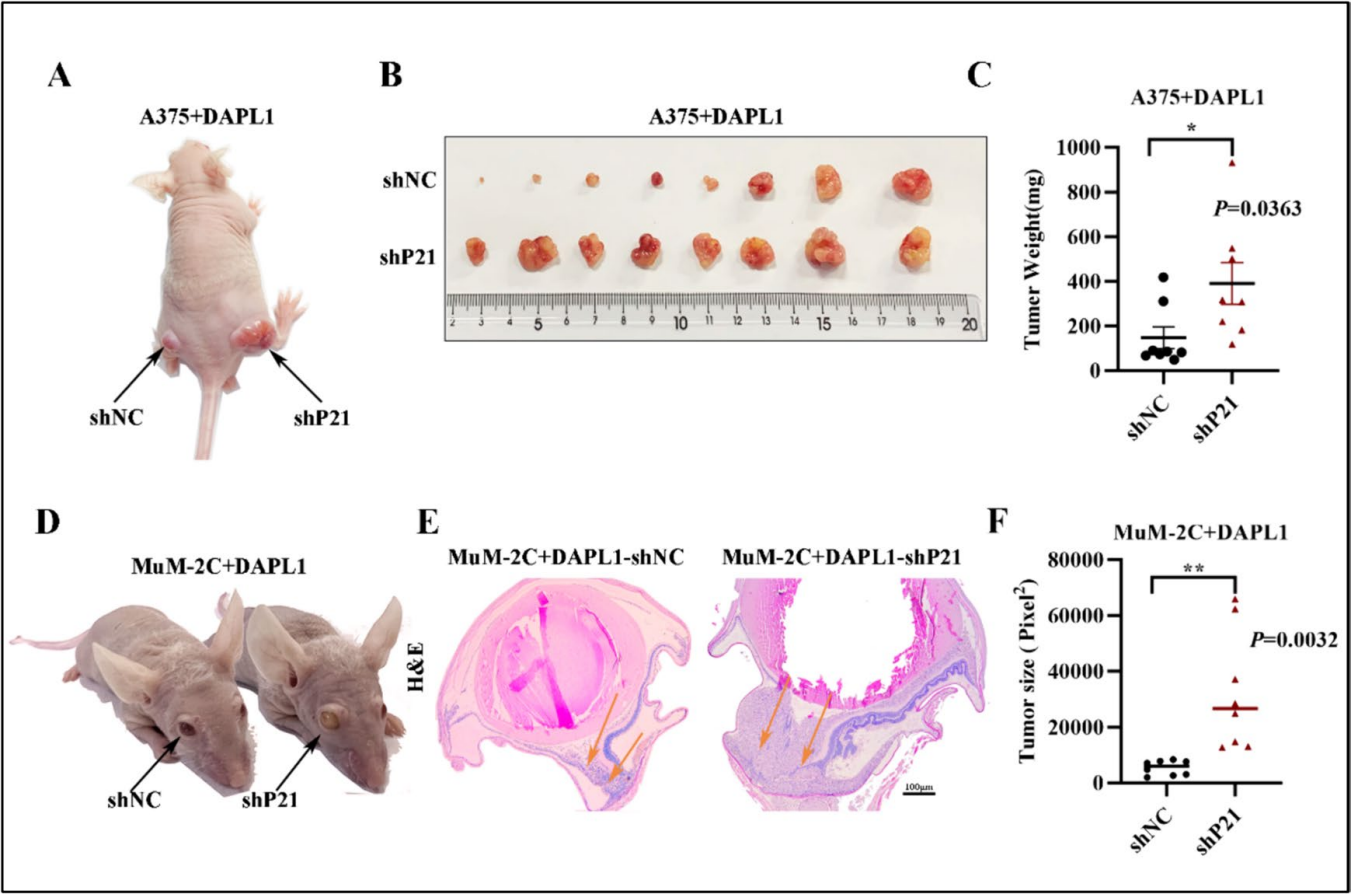
Knockdown of P21 suppresses DAPL1-dependent inhibition of melanoma tissue growth in vivo. **A** A375 + DAPL1 + shNC or A375 + DAPL1 + shP21 cells were injected subcutaneously into nude male mice (n = 8). **B** The melanoma tumors were isolated and photographed one month after the subcutaneous injection of DAPL1- or EGFP-overexpressing melanoma cells. **C** Quantification of the weight of the melanoma tissues. **D** MuM-2C + DAPL1 + shNC or MuM-2C + DAPL1 + shP21 cells were subretinal injected into the right eye of male nude mice (n = 8), the pathological features of the eyes were photographed, the bulging and protruding eyeballs could be observed in the MuM-2C + DAPL1 + shP21 cell injected mice (black arrow indicated). **E**, **F** H&E staining of the eye sections after the UM tumor formation. The ocular melanoma size of each group was quantified with Image J software. Scale bar: 100 μm. The means ± SE rpresent three independent experimental results. ** indicates p < 0.01

To determine whether P21 mediates DAPL1 inhibition of melanoma tumor growth, we constructed a shRNA lentivirus targeting P21 (shP21) to knock down P21 in A375 + DAPL1 and MuM-2C + DAPL1 cells. As the data shown, shP21 significantly decreased the protein level of P21 and increased the protein level of E2F1 in A375 + DAPL1 and MuM-2C + DAPL1 cells (Fig. 7A, B). The cell growth curves, colony formation, and EdU staining assays showed that proliferation of MuM-2C + DAPL1 cells increased after knockdown of P21 (Fig. 7C–G). In addition, we further investigated the contribution of P21 knockdown to the tumorigenesis of A375 + DAPL1 and MuM-2C + DAPL1 cells in vivo. We injected the same number of A375 + DAPL1 + shNC and A375 + DAPL1 + shP21 cells into the subcutaneous of the 6-week-old BALB/c male nude mice, and quantitated tumorigenesis one month later. As the data shown, subcutaneous xenograft tumors developed from A375 + DAPL1 + shP21 cells were larger than the shNC group tumors (Fig. 8A–C). Moreover, MuM-2C + DAPL1 + shNC and MuM-2C + DAPL1 + shP21 cells into the subretinal of the right eye of 6-week-old nude male mice, and quantitated tumorigenesis one month later. As shown in Fig. 8D, subretinal injection of MuM-2C + DAPL1 + shP21 cells caused obvious exophthalmos, consistent with a large tumor volume in the eye. Pathological analysis of eye tissue sections showed significantly more extensive tumor sizes with MuM-2C + DAPL1 + shP21 melanoma compared to MuM-2C + DAPL1 + shNC mice (Fig. 8E, F), indicating that knockdown of P21 attenuates the inhibitory effect of DAPL1 on melanoma tumor growth.

## Discussion

**Fig. 9:**
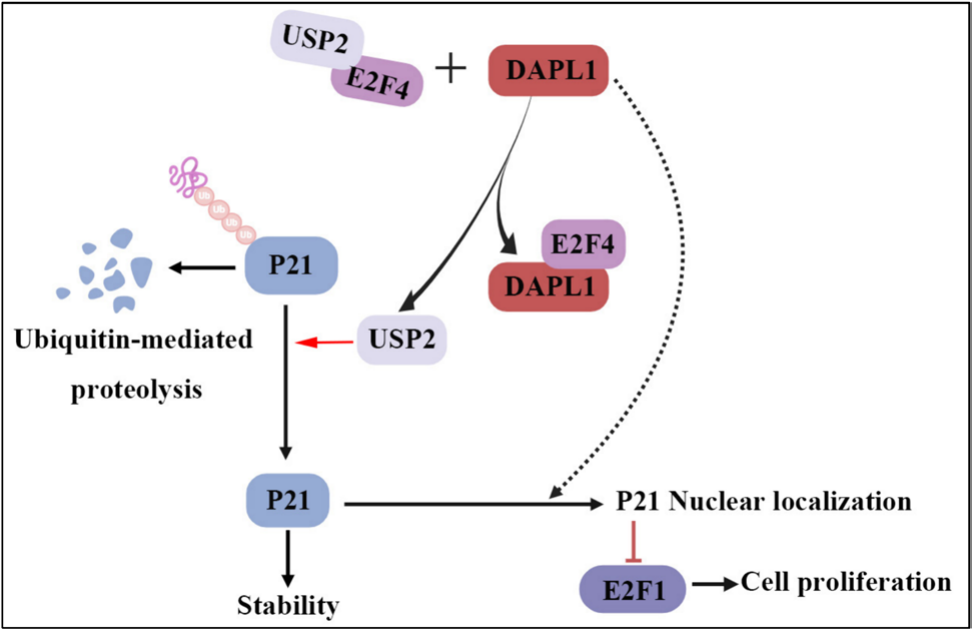
Diagram illustrating role(s) of DAPL1 acts as a melanoma cell proliferation inhibitor through increasing the stability of P21 protein. In this model, DAPL1 increases the protein level of P21 by promoting the deubiquitination of the P21 protein to inhibit the ubiquitination dependent degradation and thereby promoting its stability in melanoma cells, thus inhibiting the cell proliferation and tumor growth

Malignant melanoma is a highly lethal tumor, while molecular mechanisms underlying melanoma pathogenesis have not been fully understood, a more thorough understanding of this process might lead to new treatments and better outcomes for patients with this deadly cancer. In this study, we used bioinformatics analysis, cellular and biochemical investigations, and a nude mouse model, all of which consistently support the role of DAPL1 as a novel melanoma suppressor gene. First, Both of CM and UM cells express low level of DAPL1. Second, UM patients expressing low DAPL1 levels have an unfavorable prognosis. Third, overexpression of DAPL1 inhibits melanoma cell proliferation in culture and inhibits melanoma tumor growth in nude mice. Fourth, DAPL1 decreases turnover of P21 in melanoma cells, and knockdown of P21 neutralizes inhibition of melanoma cell proliferation by DAPL1. The molecular pathway through which this occurs appears to be: DAPL1 → decreased P21 ubiquitination → increased P21 stability → decreased melanoma cell proliferation (schematically illustrated in Fig. 9), which is probably only one of multiple pathways involved in regulating melanoma tumorigenesis.

DAPL1 has received relatively limited attention in functional and clinical studies. It was initially speculated to be involved in epithelial cell differentiation and apoptotic processes [1]. More recently, additional functions of DAPL1 have been identified, including roles in T cell immunity, mRNA translational inhibition, and age-related macular degeneration (AMD), a leading cause of irreversible blinding disease in the aged population [7, 10, 24]. Our previous work demonstrated that DAPL1 plays critical functional roles in maintaining RPE (retinal pigmented epithelium) cell homeostasis and antioxidation [8, 10, 17]. Here we further show that DAPL1 regulates melanoma tumorigenesis, suggesting that DAPL1 has multiple critical biological functions. Further work is needed to clarify the roles of DAPL1 in physiological and pathological conditions.

Cell proliferation normally is a precisely controlled cellular event, while uncontrolled cell hyperproliferation is a critical causing factor of tumorigenesis. Previous reports have shown that DAPL1 regulates proliferation of fetal pituitary cells [4] and that DAPL1 inhibits proliferation of RPE cells by up-regulating the level of P21 [9, 17]. In addition, we have shown that DAPL1 promotes P21 phosphorylation and stabilization in RPE cells to regulate RPE cell EMT [8]. However, the mechanism by which DAPL1 regulates P21 protein levels had not been elucidated. Consistent with these findings, in this work we show that DAPL1 does not affect the mRNA level of P21 in melanoma cells but instead increases the stability of P21 protein through decreasing its ubiquitination to decrease its turnover. Thus, DAPL1 regulates P21 protein levels through post-transcriptional regulation, although the precise mechanisms vary slightly in different cell types.

P21 is a cyclin-dependent kinase (CDK) inhibitor whose stability is essential for proper cell cycle progression. It has been shown that ubiquitination plays a crucial role in regulating stability of P21 protein [23]. Aberrant function of the ubiquitination system leads to several disorders, including cancer [25]. Degradation of P21 by proteasomes is a potential mechanism of regulating of the P21 protein levels [26], and several genes have been reported to regulate P21 stability by mediating its ubiquitylation [9, 27, 28]. Here, we show that DAPL1 acts as a novel P21 ubiquitylation regulator in melanoma cells, which deepens our understanding of this process. However, the precise mechanisms through which DAPL1 regulates the ubiquitination of P21 remains to be elucidated, and further investigation is needed to address this critical question. In fact, our previous work reported that DAPL1 could bind directly with E2F4 in RPE cells [10]. It has been reported that E2F4 binds directly with USP2 (ubiquitin specific peptidase 2) in gastric cancer cells [29], while USP2 could bind with SKP2 and USP2-stabilized SKP2 did not destabilize its substrate P21 [30]. Based on a combination of these reports we hypothesized that DAPL1 regulation of P21 ubiquitination might partially be through interaction with the E2F4/USP2 complex.

CD8 + T cells play critical roles in the immune response against infections and cancer. Previous reports have shown dysfunctional CD8 + T cells can form a proliferative, dynamically regulated compartment within human melanoma [31]. Exhausted CD8 + T cells include a subpopulation of ‘progenitor-exhausted’ cells, which can respond to anti-PD-1 therapy. Melanoma patients with a higher percentage of progenitor-exhausted cells experience a longer duration of response to checkpoint-blockade treatment [32]. NFATc2 (nuclear factor of activated T cells 2) is reported to be an intrinsic regulator of melanoma dedifferentiation [33]. Intriguingly, DAPL1 controls NFATc2 activation to regulate CD8 + T cell exhaustion and responses in chronic infection and cancer, including melanoma [7]. Thus, besides the results presented this work, the complex functional roles of DAPL1 in melanoma tumorigenesis might include immunity and inflammation among others. Clarification of the precise mechanisms of DAPL1 in melanoma tumorigenesis will deepen our understanding of the pathogenesis of melanoma and potentially provide an actionable therapeutic strategy, which needs to be addressed in future work.

In summary, our study reveals a novel mechanism in which DAPL1 acts as a tumor suppressor by inhibiting the proliferation of melanoma cells by increasing P21 protein stability. Our findings present a new perspective for understanding the development of melanoma and suggests potential targets for treating this deadly cancer.

## Supplementary Information

Below is the link to the electronic supplementary material.

Supplementary file1 (PDF 2465 KB)

## Data Availability

The datasets used and/or analyzed during the present study are available from the corresponding author on reasonable request.
