# Supplementary material for: Death associated protein like 1 acts as a novel tumor suppressor in melanoma by increasing the stability of P21 protein
Source: Mol Cell Biochem. 2024 Jul 9;480(3):1595–610. doi: 10.1007/s11010-024-05067-0 (PMC11842415; doi:10.1007/s11010-024-05067-0)

Fig2-B:1-1-DAPL1-A375

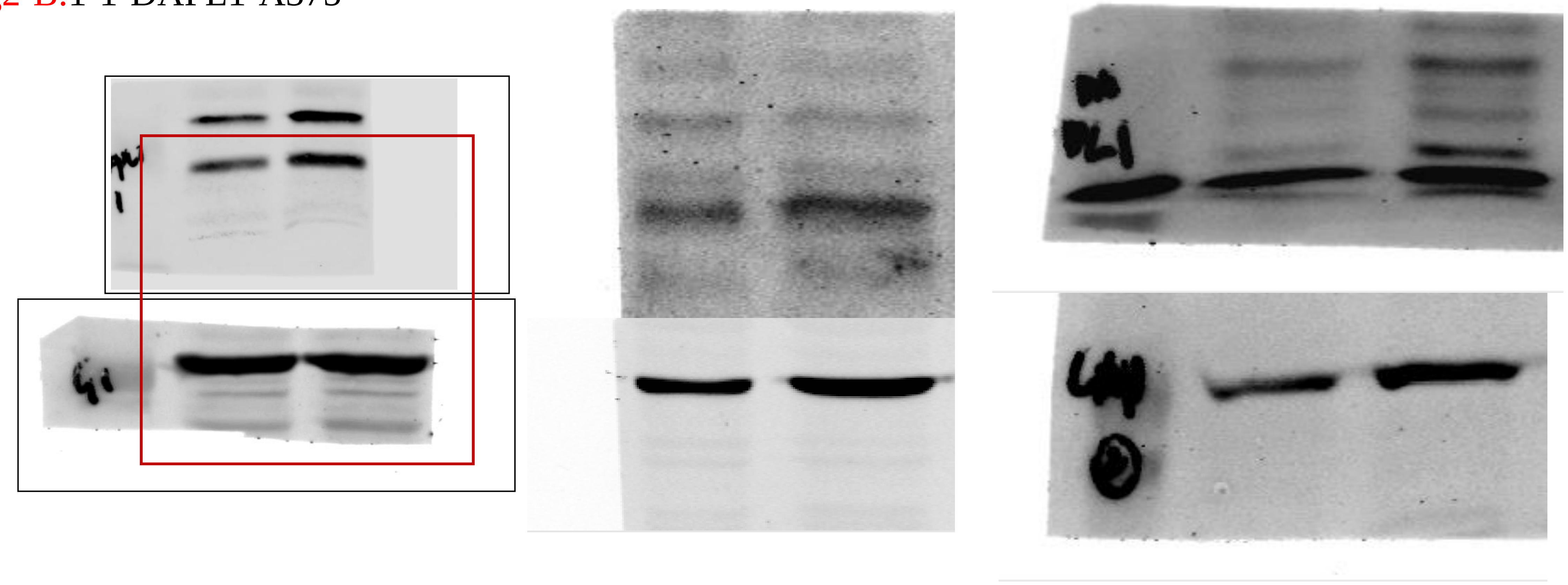

Fig2-B:1-1-DAPL1-C918

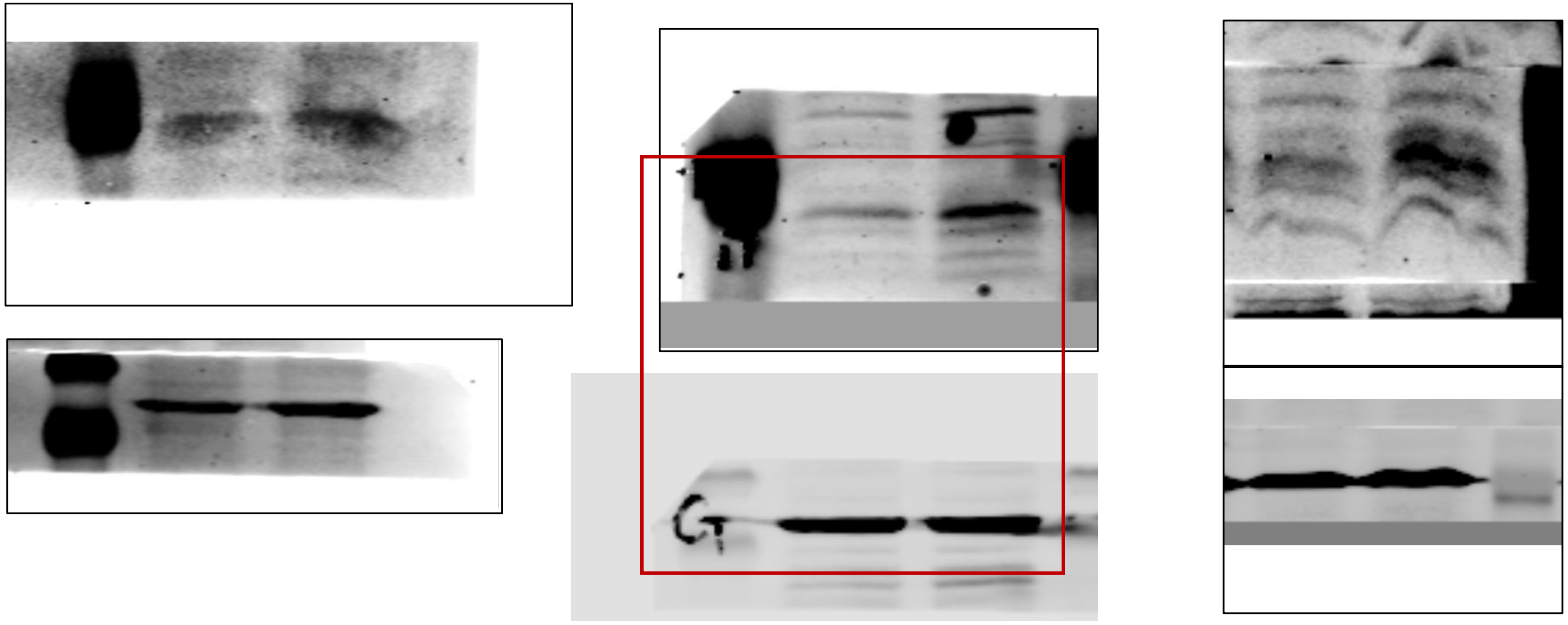

Fig2-B: 1-2-DAPL1-MuM-2C

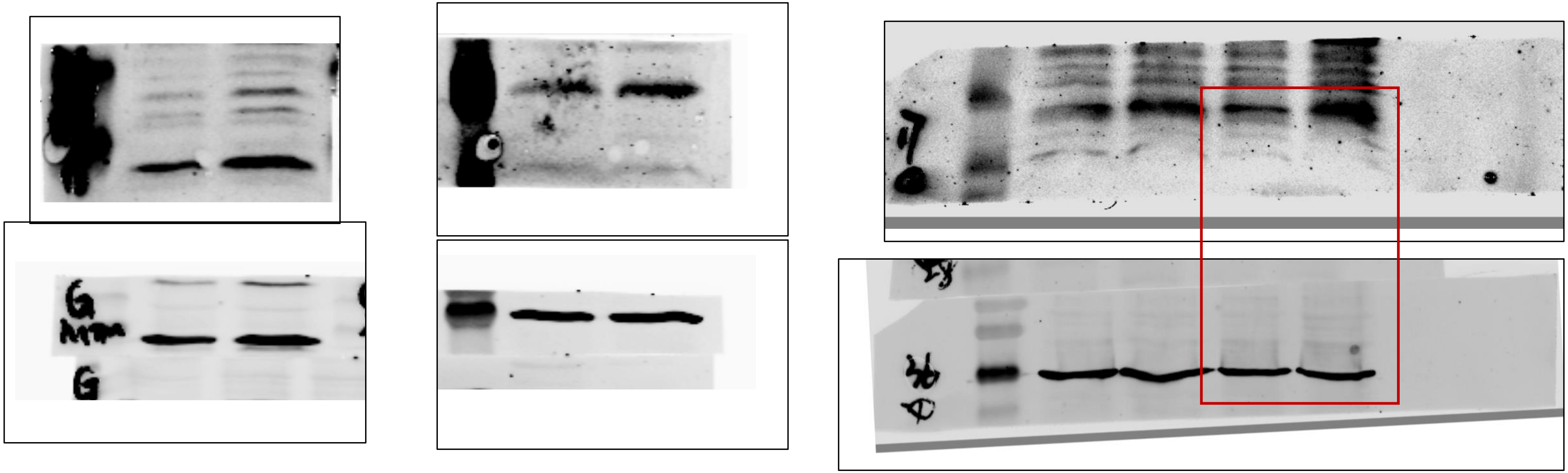

Fig3-A: 2-1-C918 si-DAPL1

DAPL1

GAPDH

P21

GAPDH

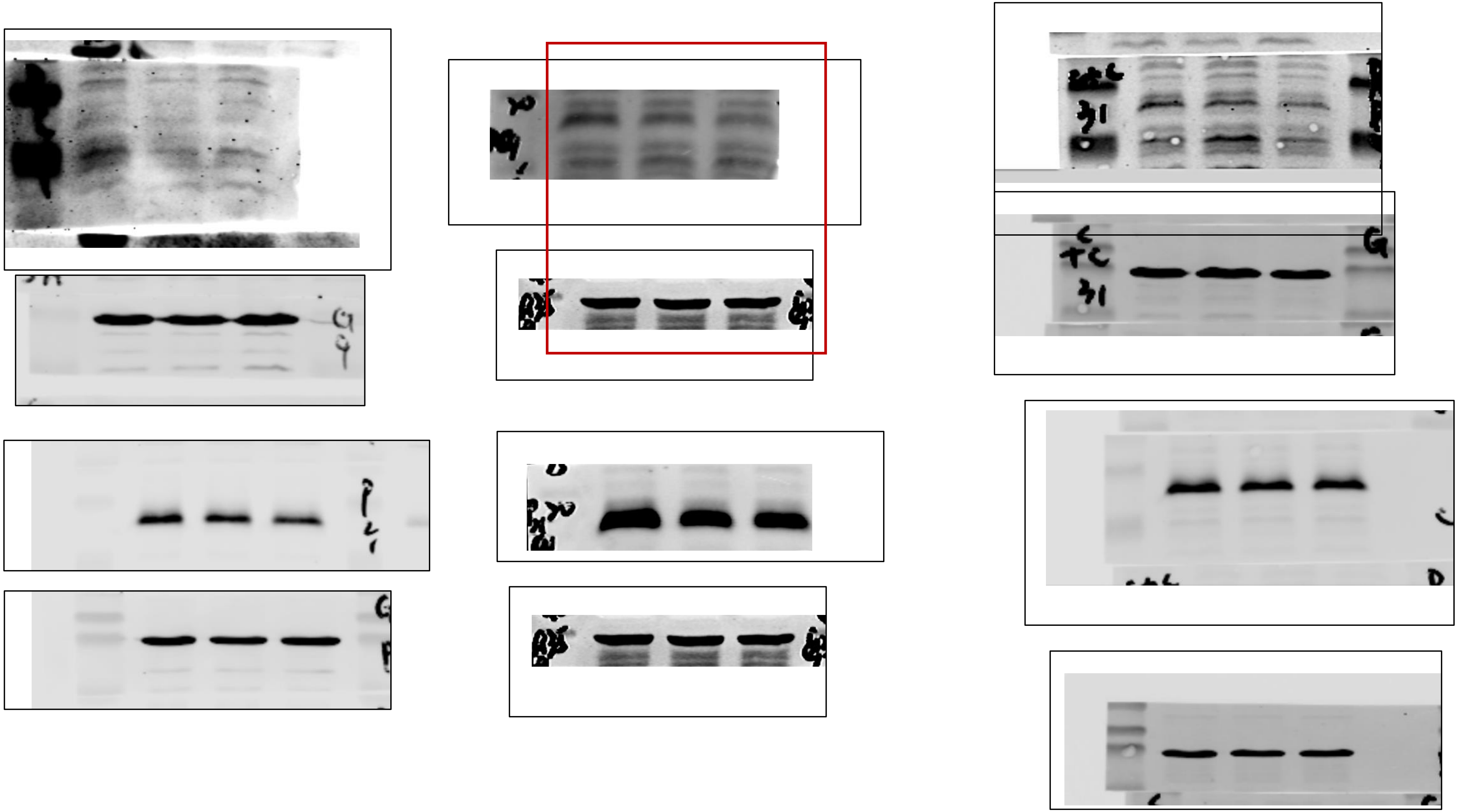

Fig3-A: **2-1-C918 si-DAPL1**

DAPL1

GAPDH

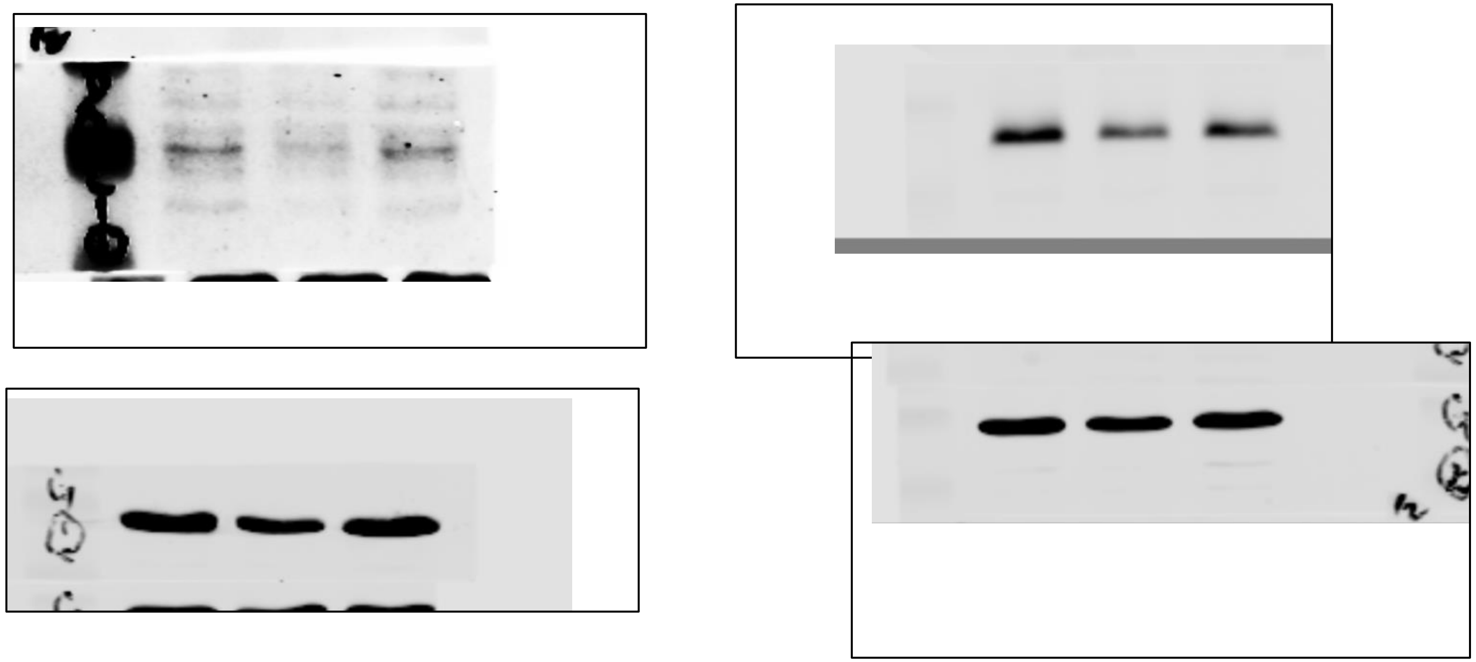

Fig3-A: 2-2-MuM-2C si-DAPL1

DAPL1

GAPDH

P21

GAPDH

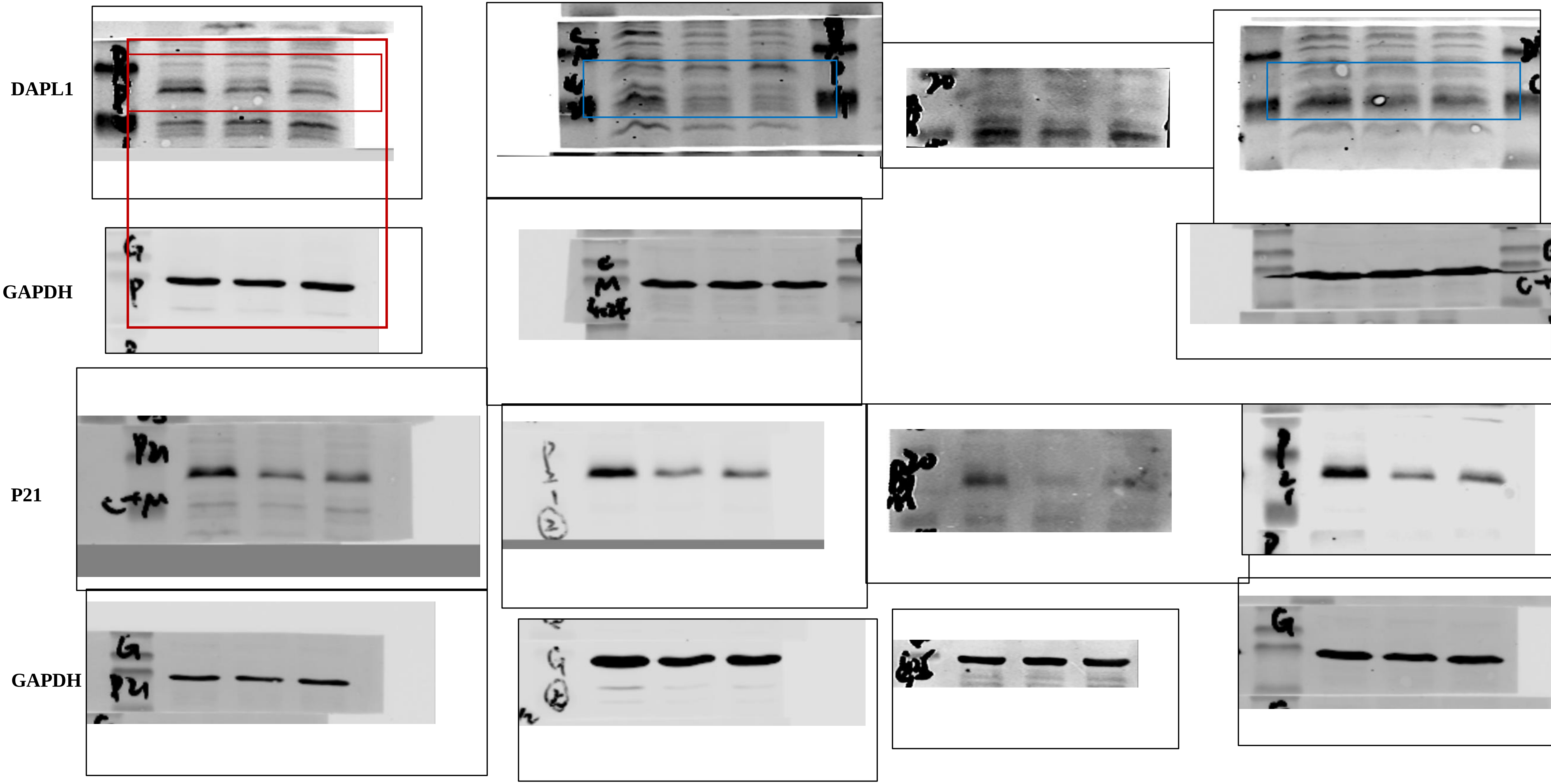

Fig5-A :3-1-P21上调-A375

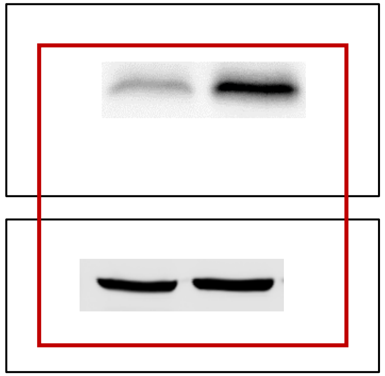

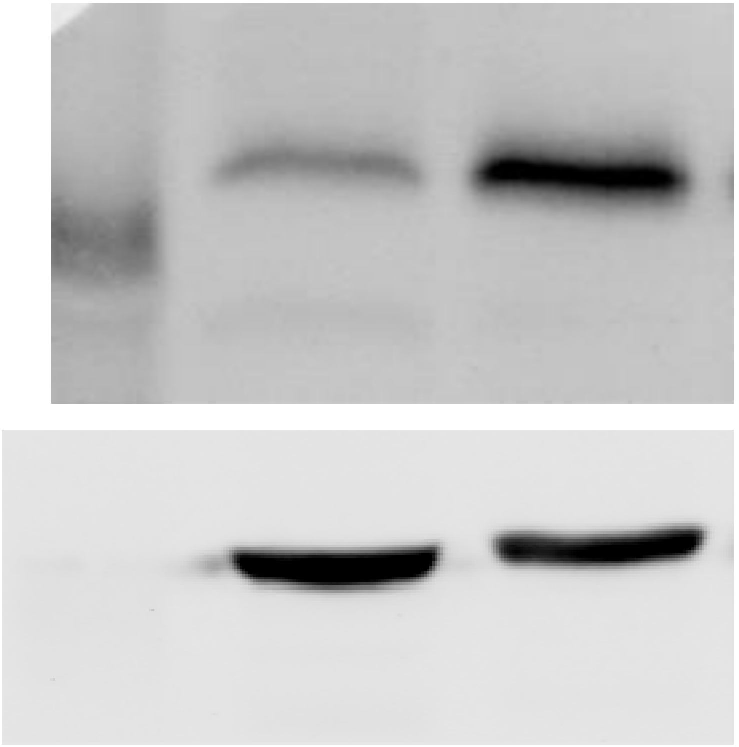

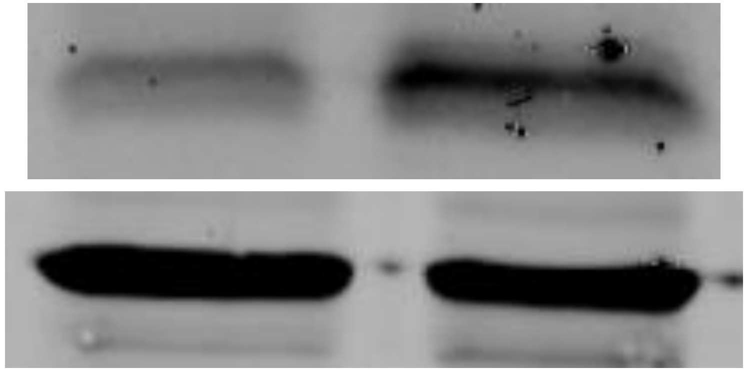

Fig5-A :3-1-P21上调-C918

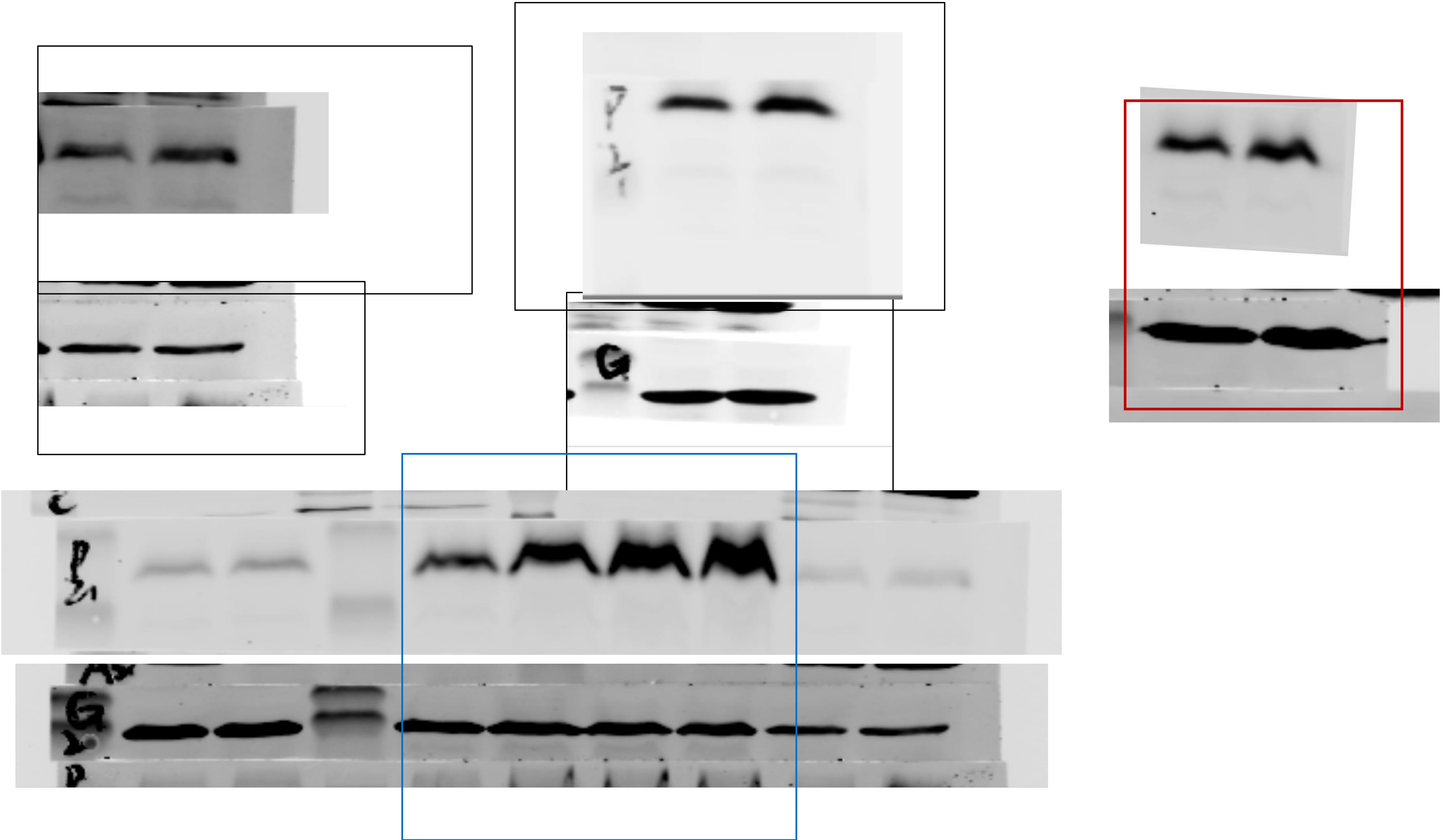

Fig5-A : 3-2-P21上调-MuM-2C

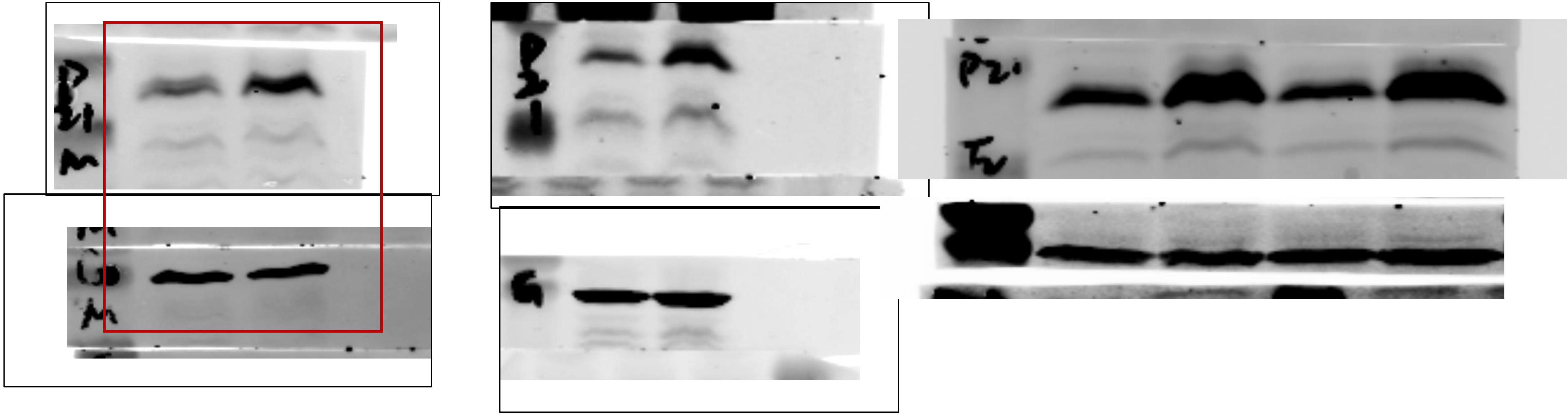

Fig5-C：4-1-核质分离-C918

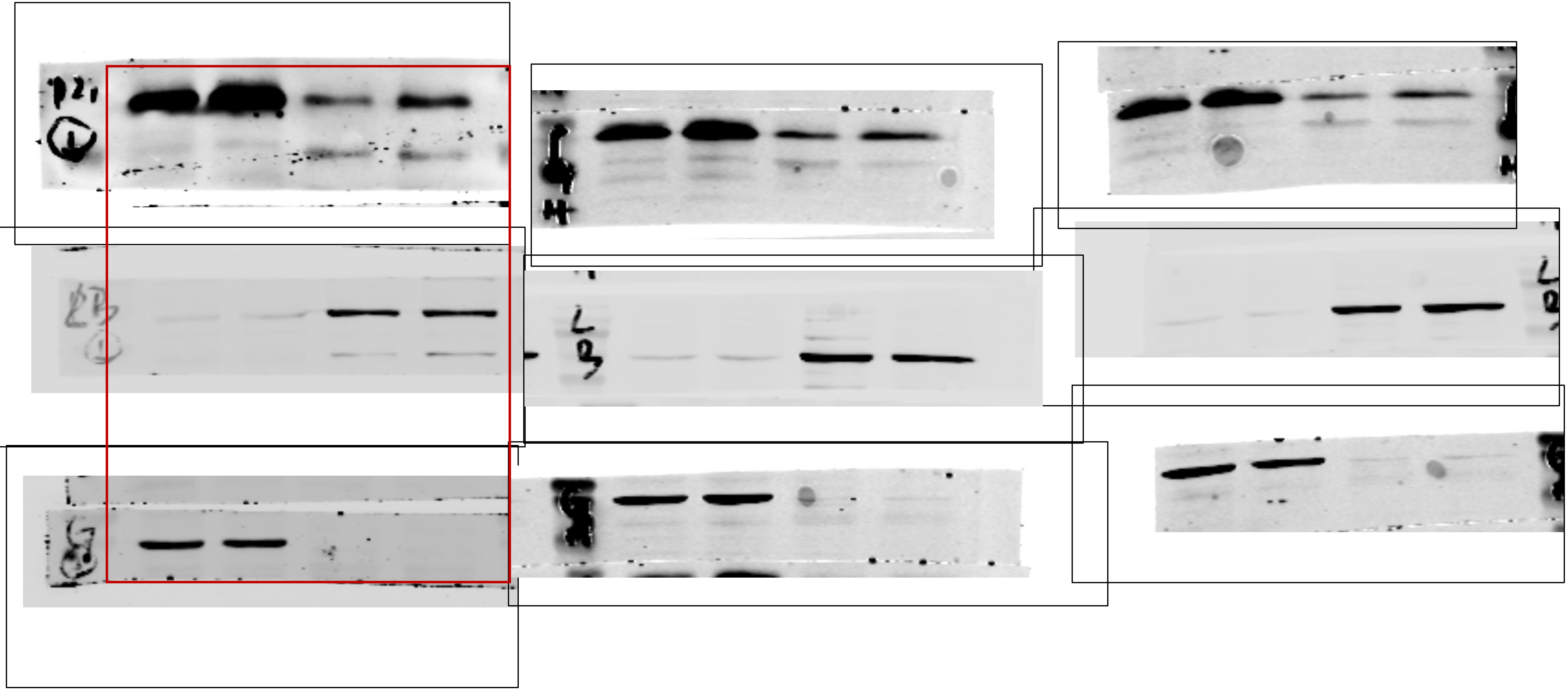

Fig5-C : 4-2-核质分离-MuM-2C

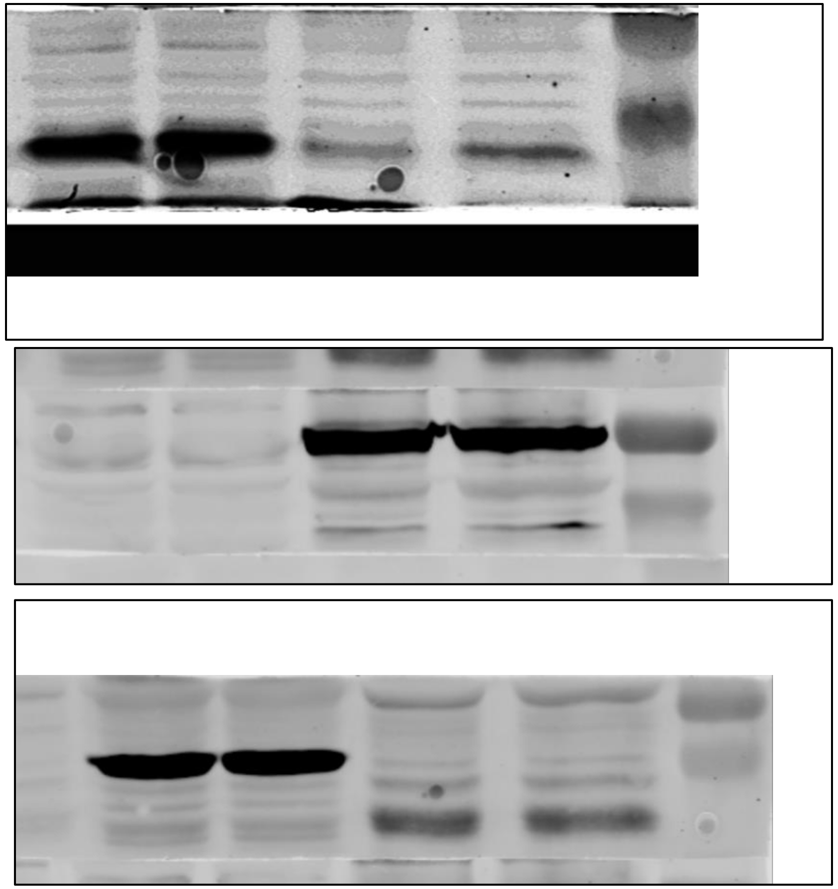

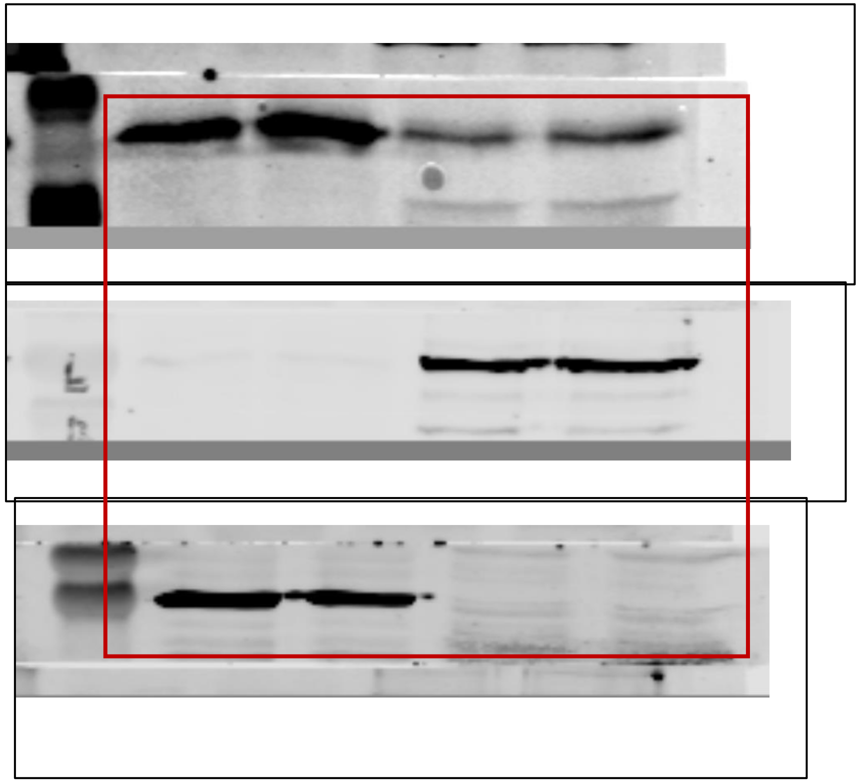

Fig5-C : 4-2-核质分离-MuM-2C

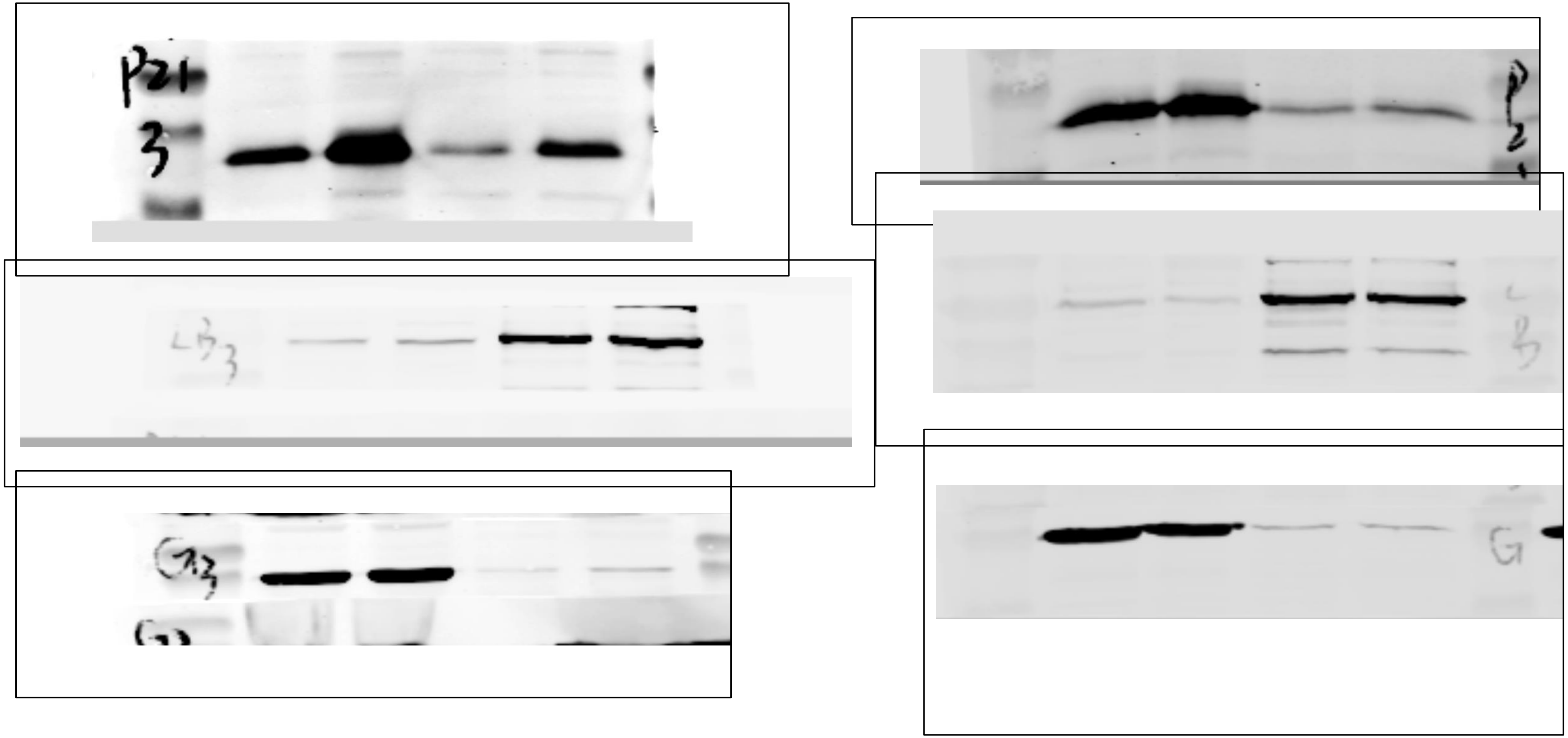

Fig5-E : 5-1-E2F1-C918

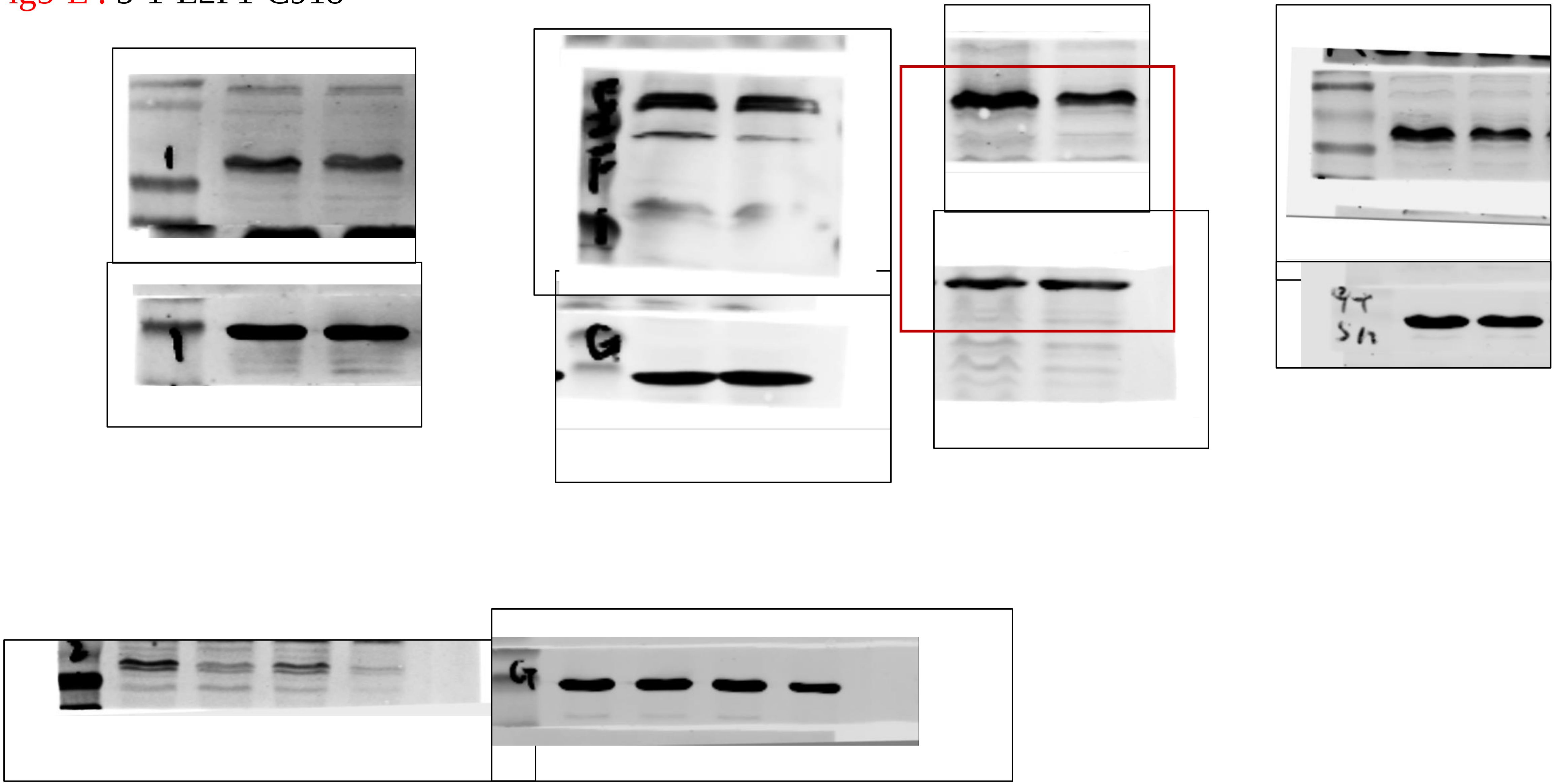

Fig5-E : 5-2-E2F1-MuM-2C

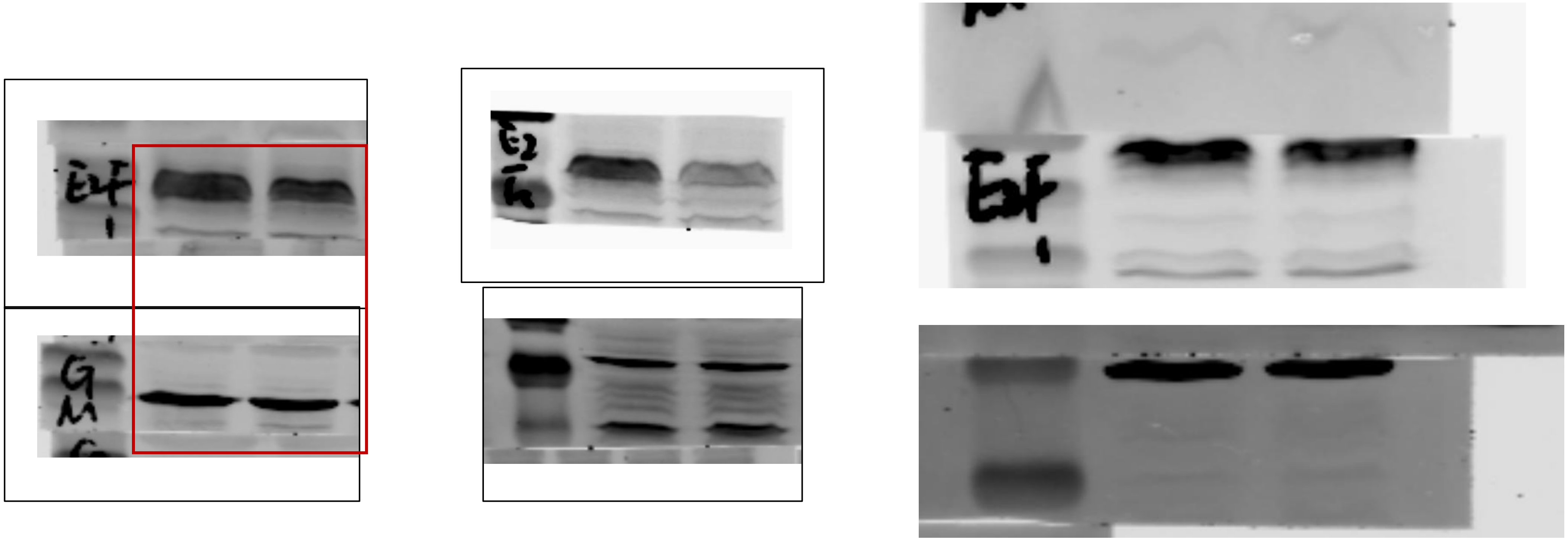

Fig6-C : **6-1-C918 CHX**

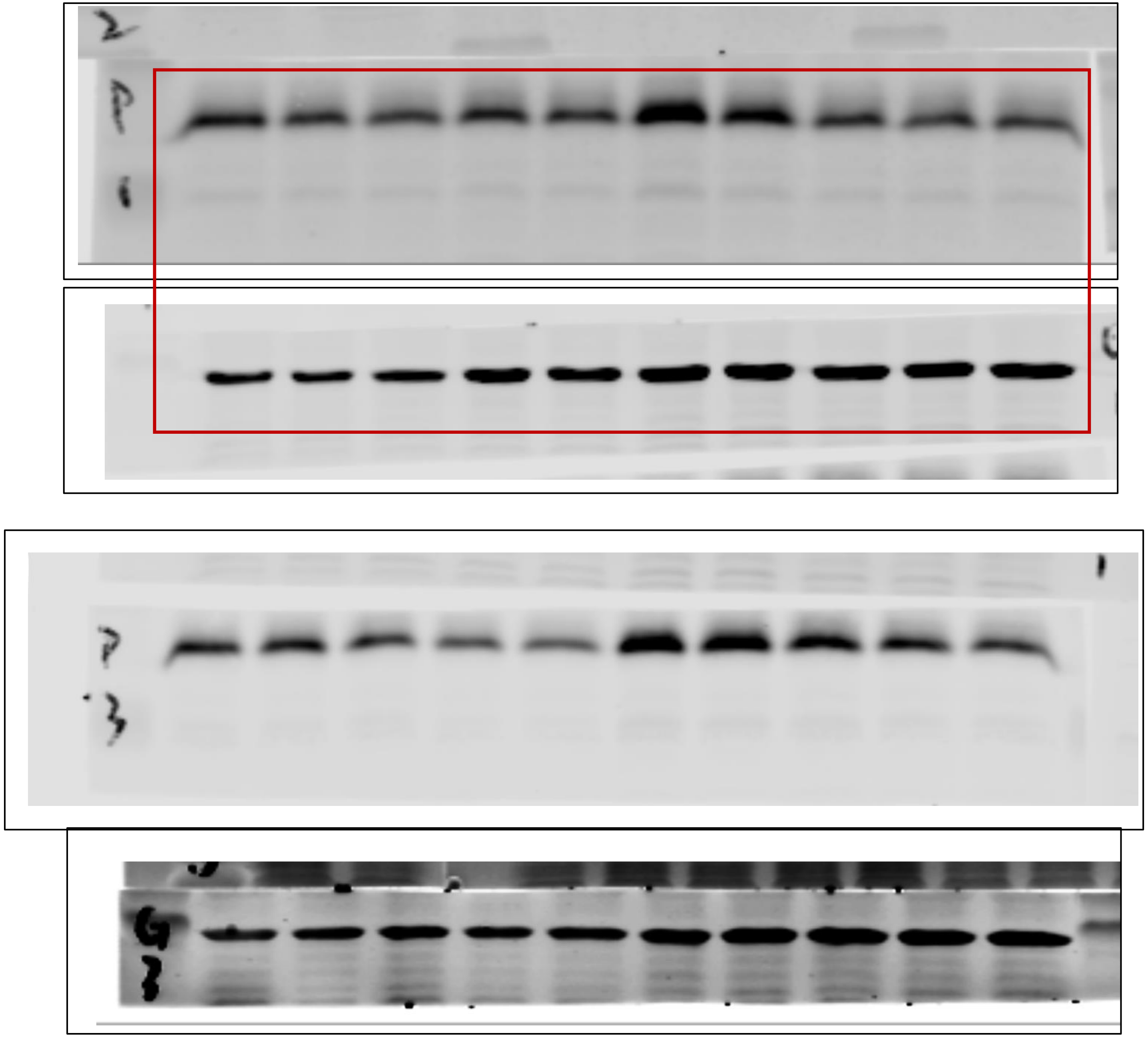

Fig6-C : **6-1-C918 CHX**

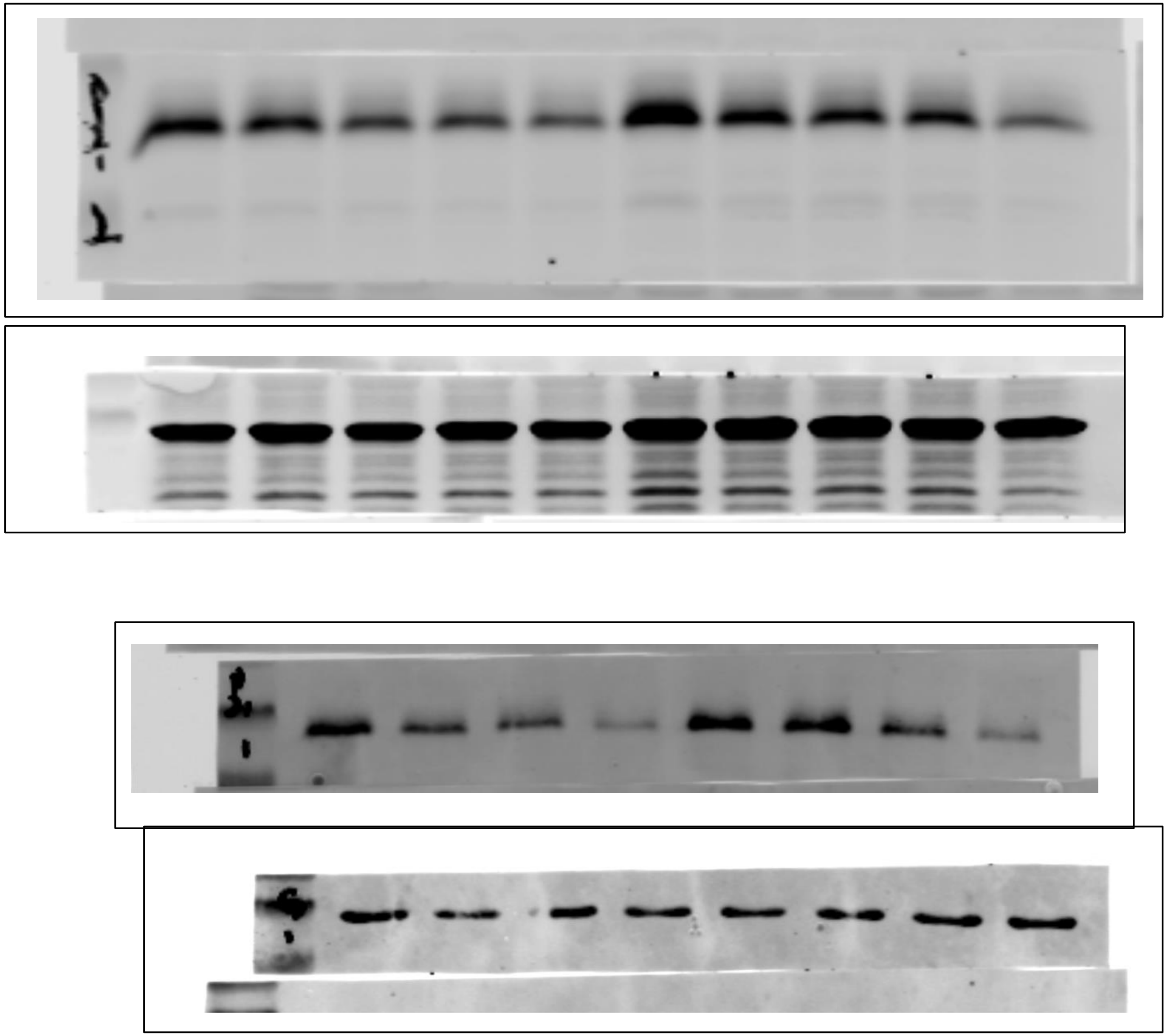

Fig6-C : **6-1-C918　CHX**

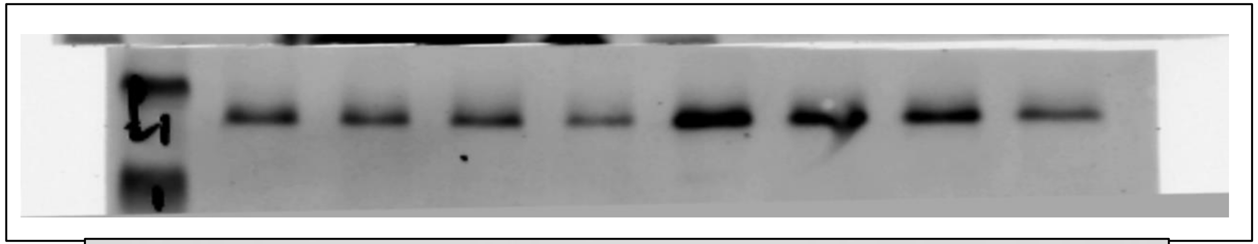

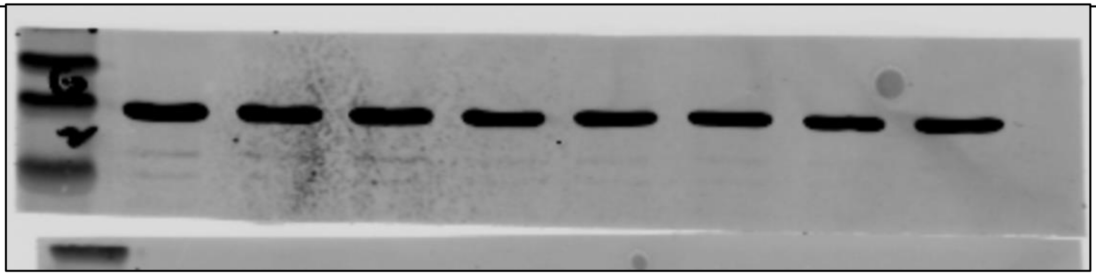

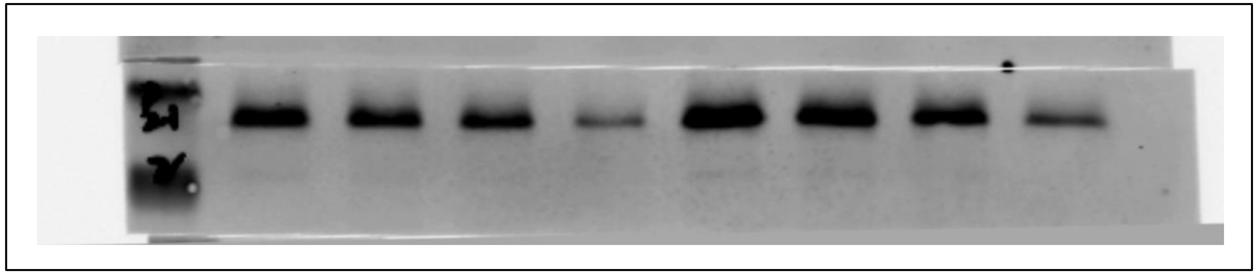

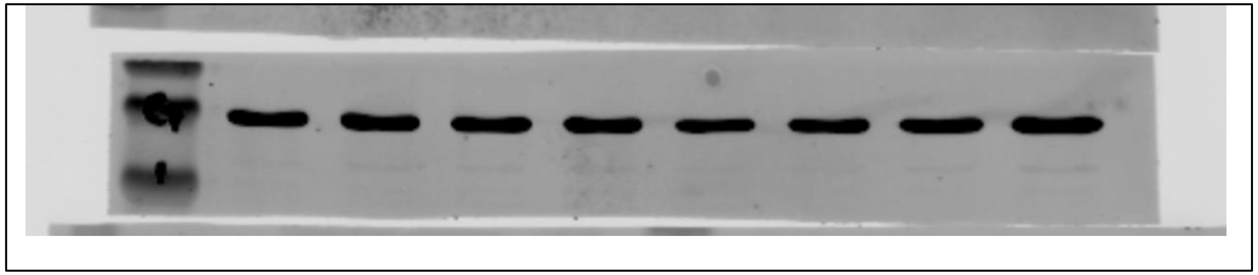

Fig6-C : **6-2-MuM-2C   CHX**

P21

GAPDH

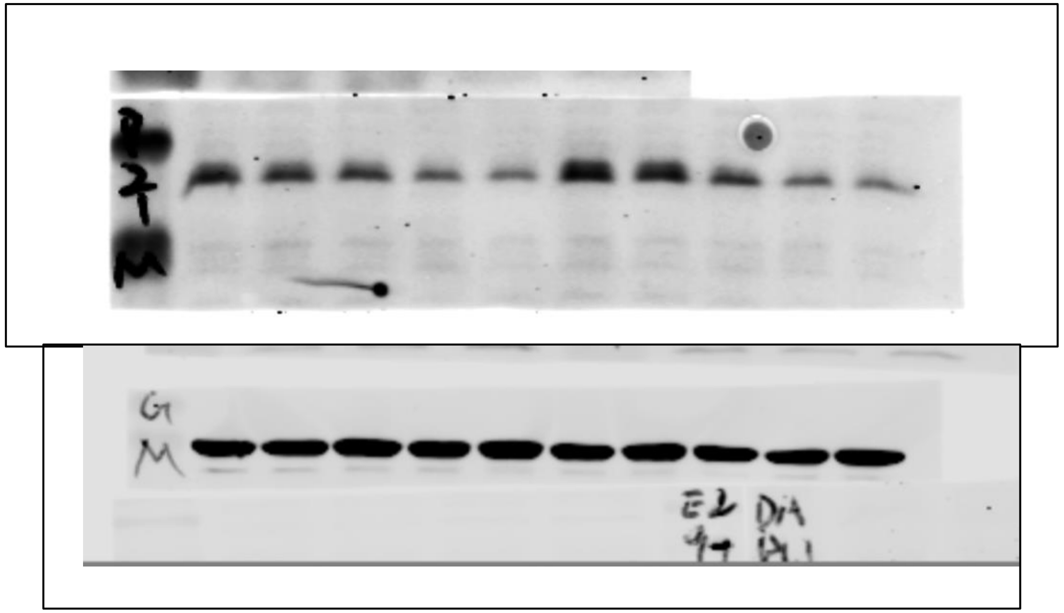

Fig6-C : **6-2-MuM-2C CHX**

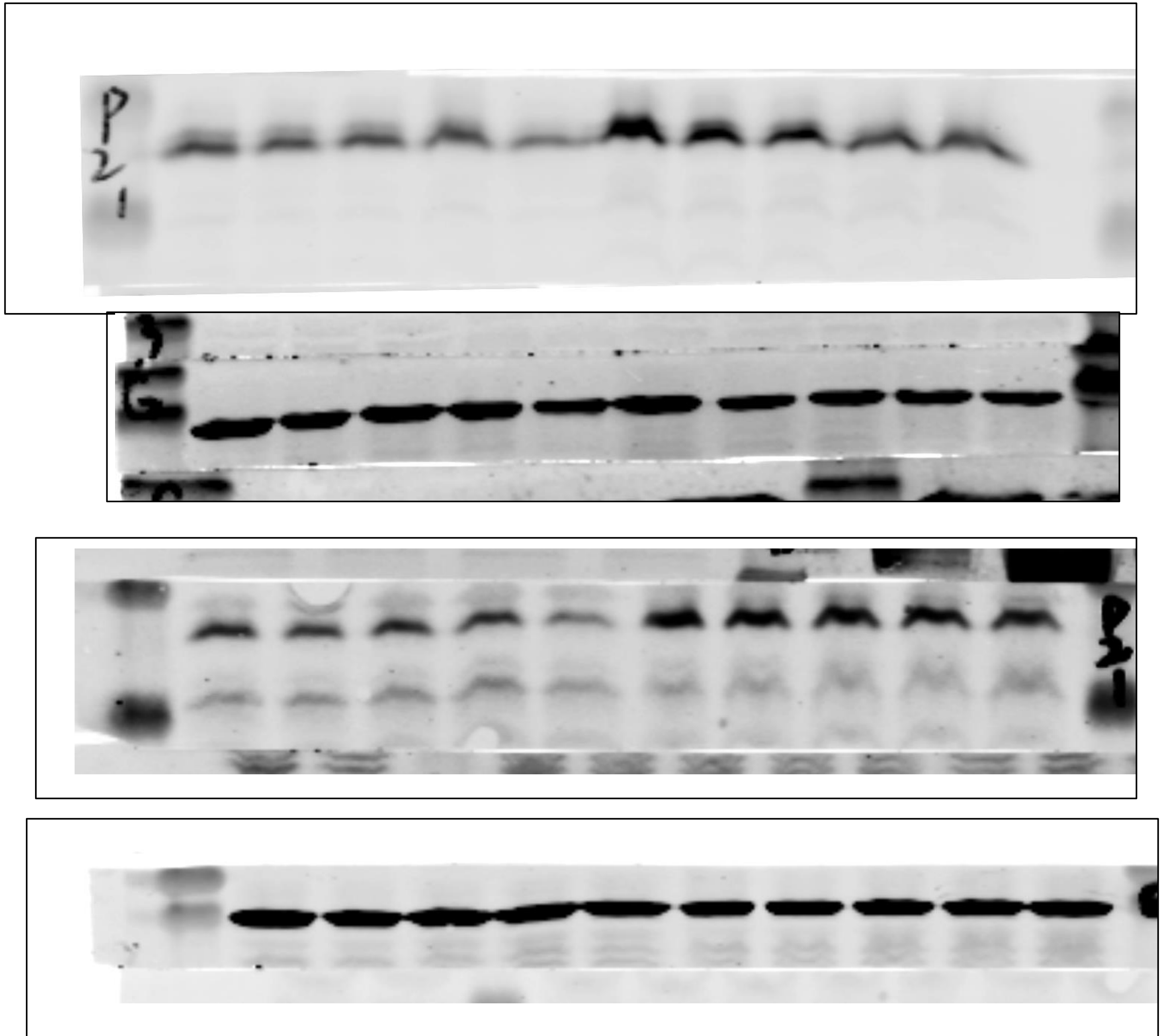

Fig6-C : **6-2-MuM-2C CHX**

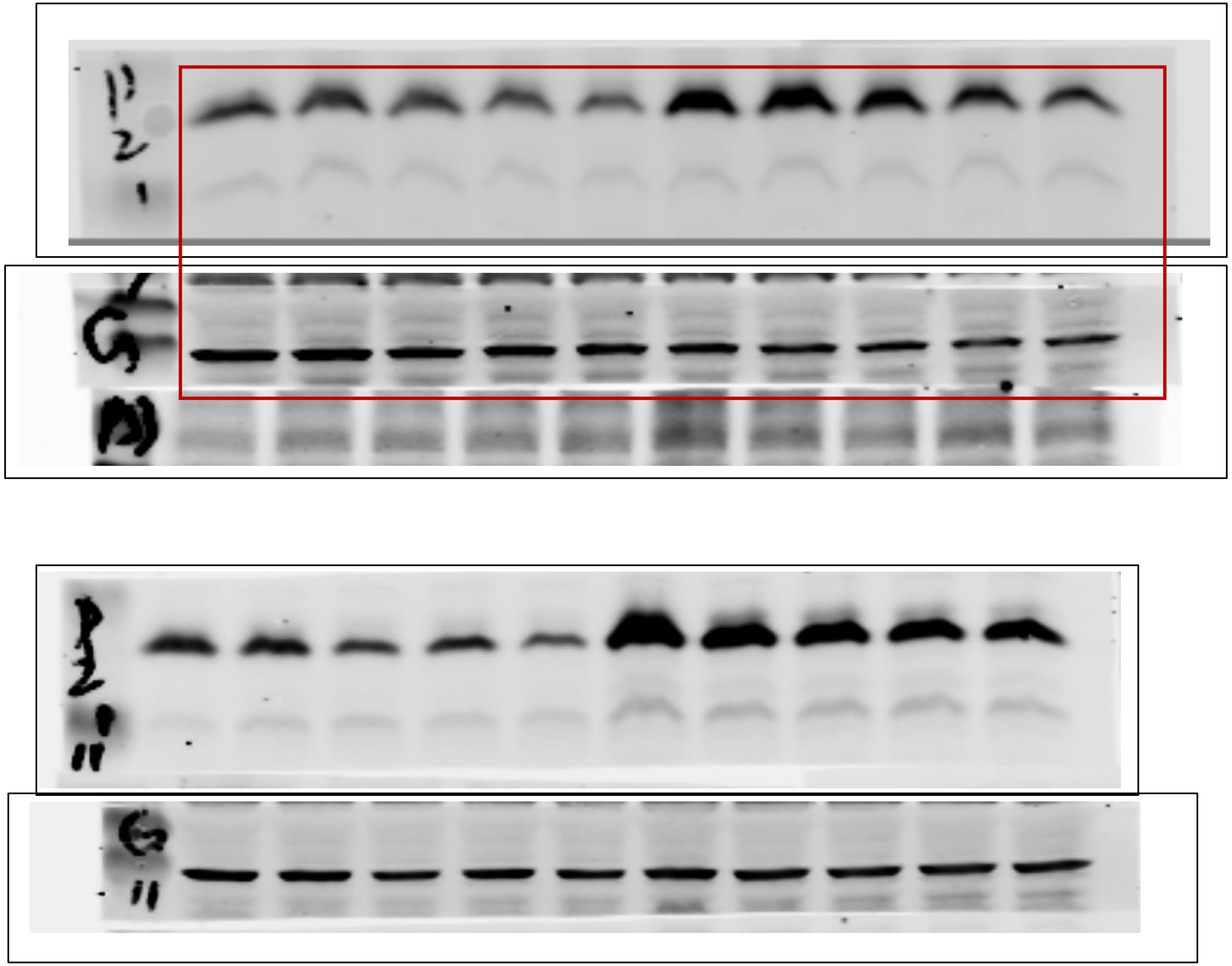

Fig6-E : 7-1-MG132-C918

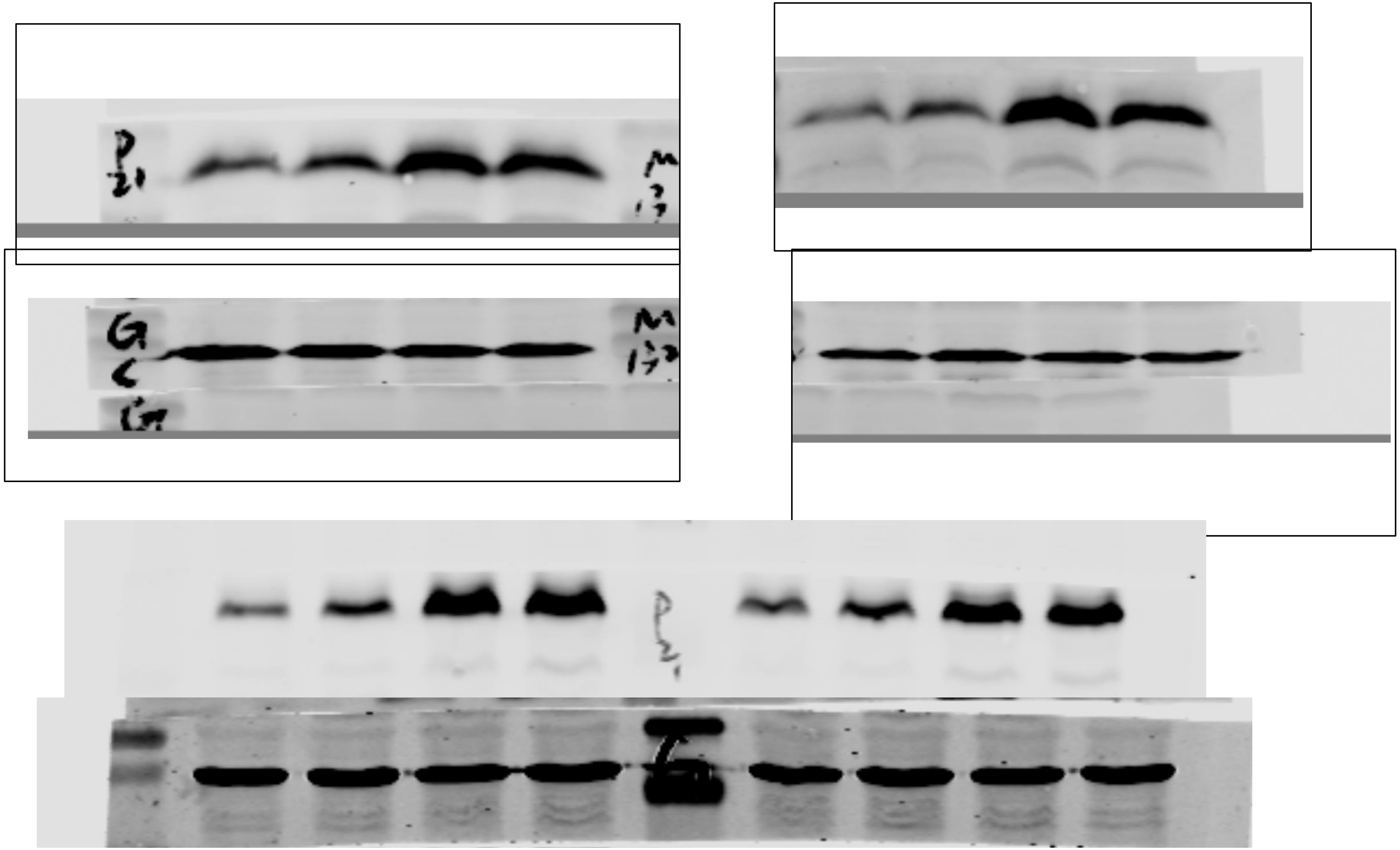

Fig6-E : 7-1-MG132- C918

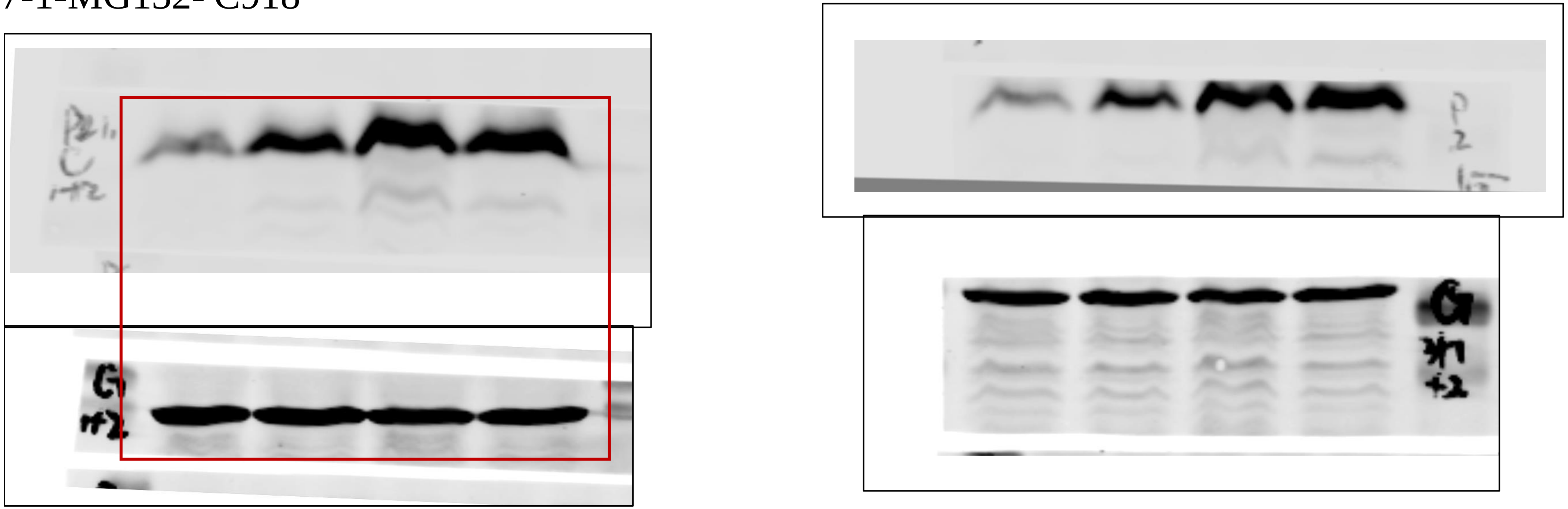

Fig6-E : 7-2-MG132- MuM-2C

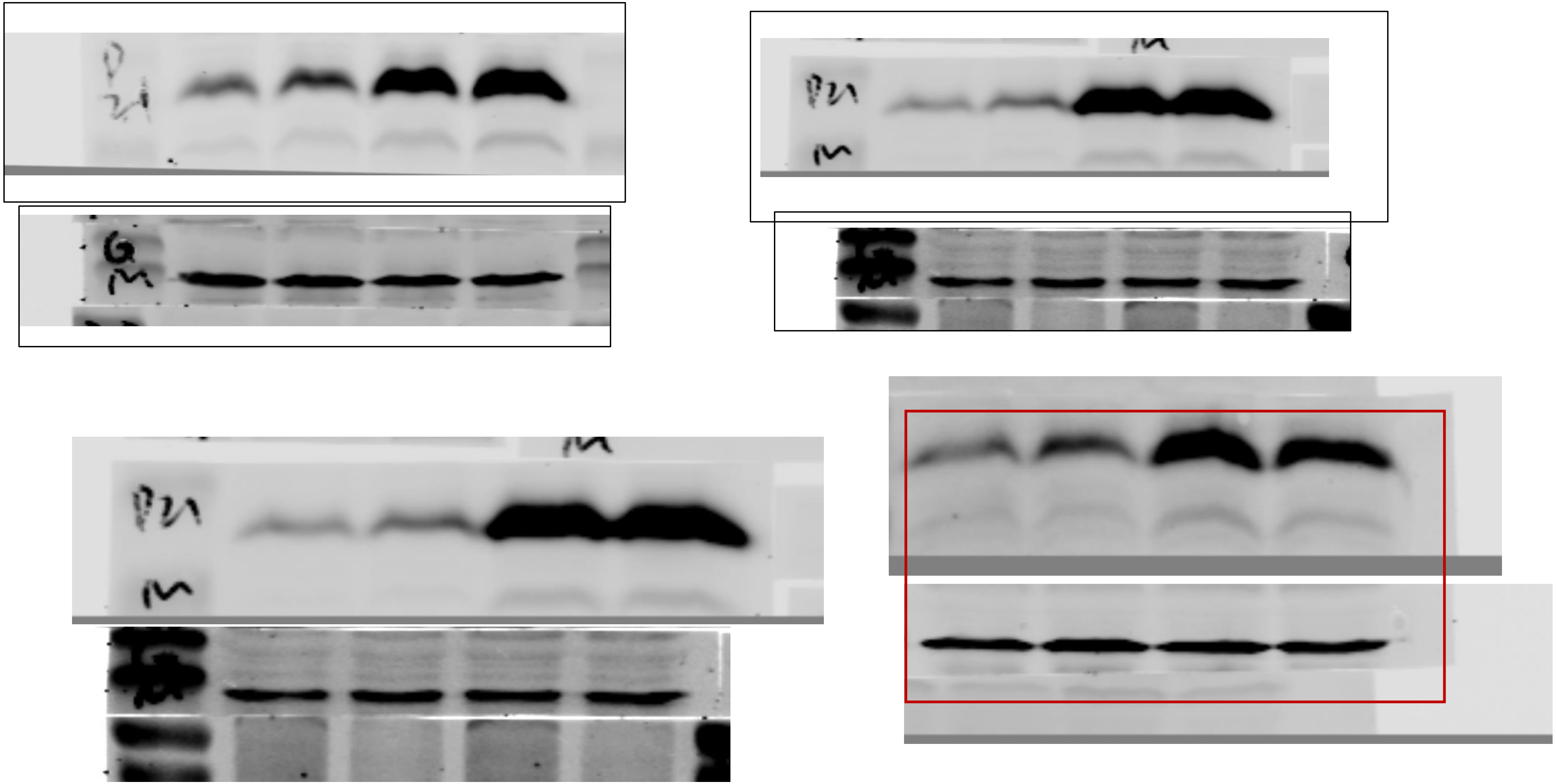

Fig6-G: **8-1-C918 UB**

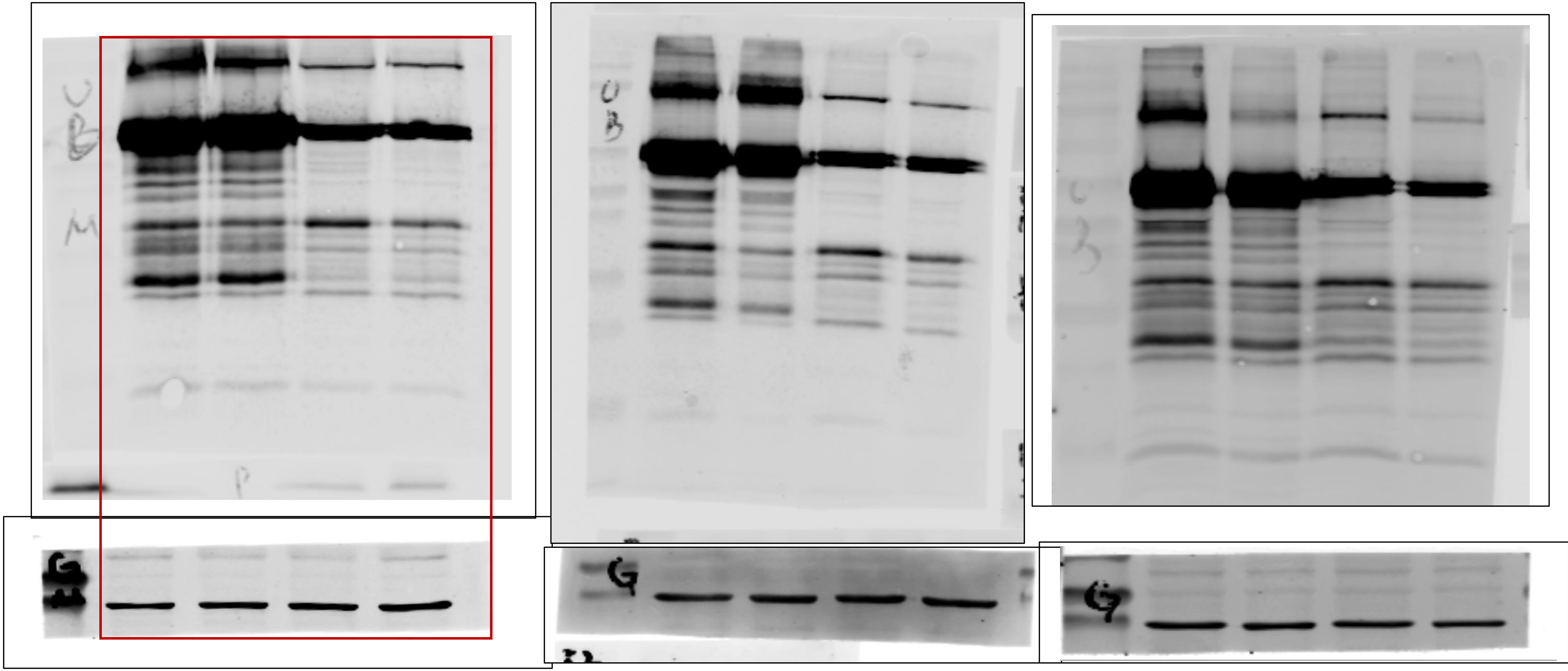

Fig6-G: **8-1-C918 UB**

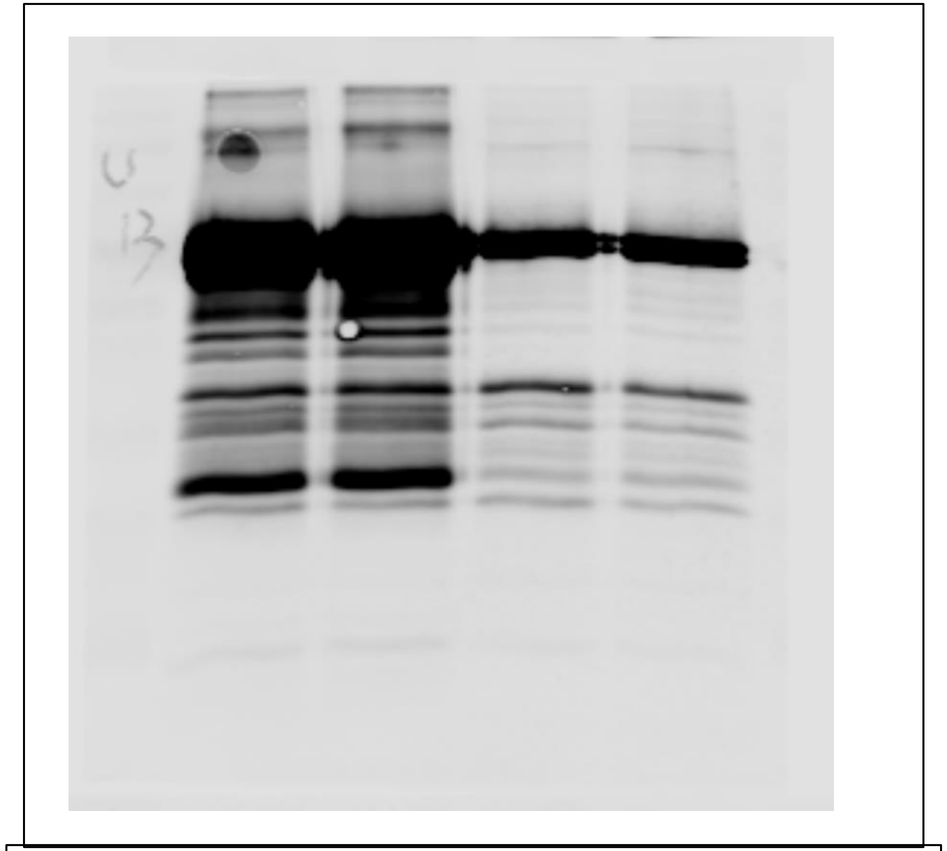

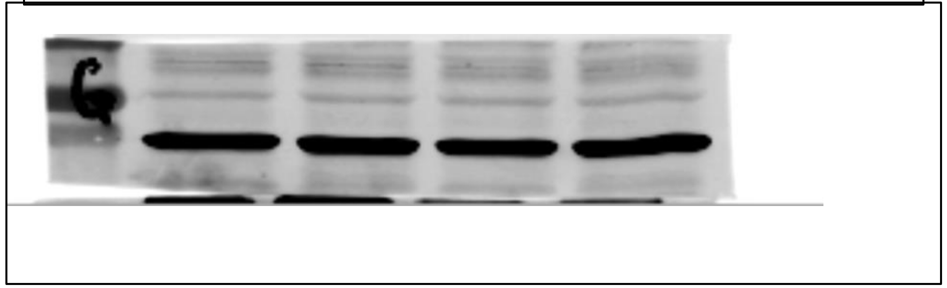

Fig6-G: **8-2-MuM-2C UB**

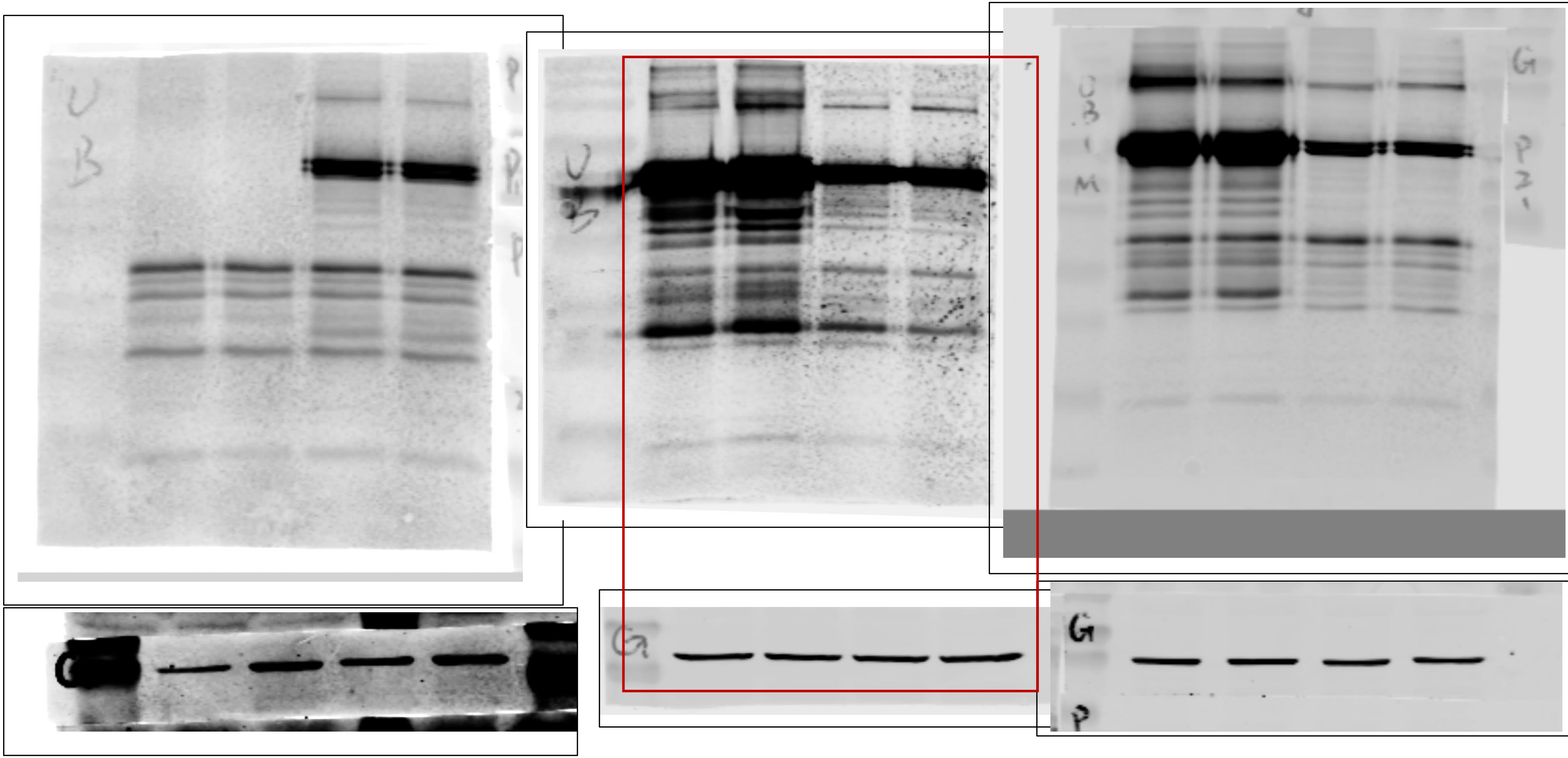

Fig6-G: **8-2-MuM-2C UB**

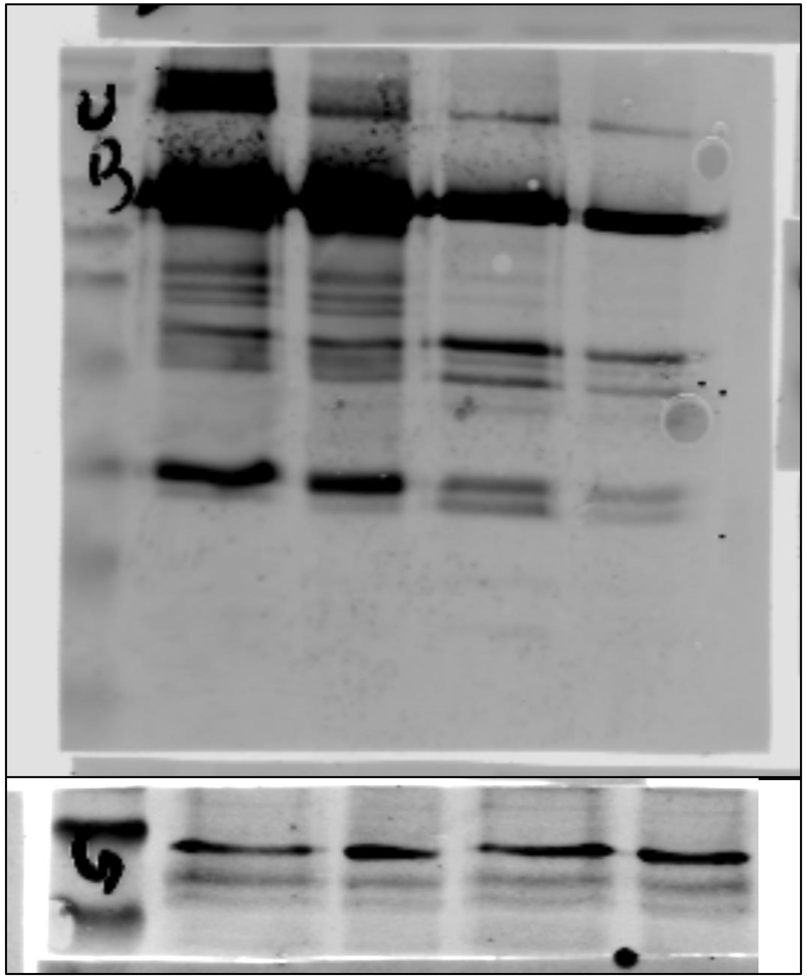

Fig7-A: **A375+DAPL1-shP21**

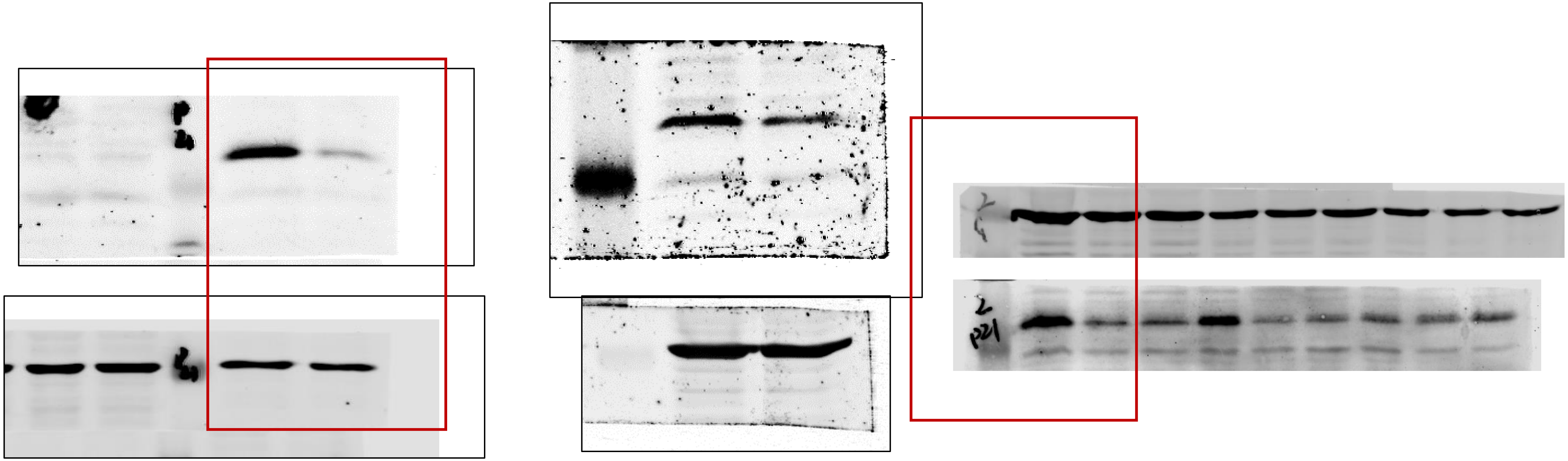

Fig7-A: **9-1-MuM-2C+DAPL1-shP21**

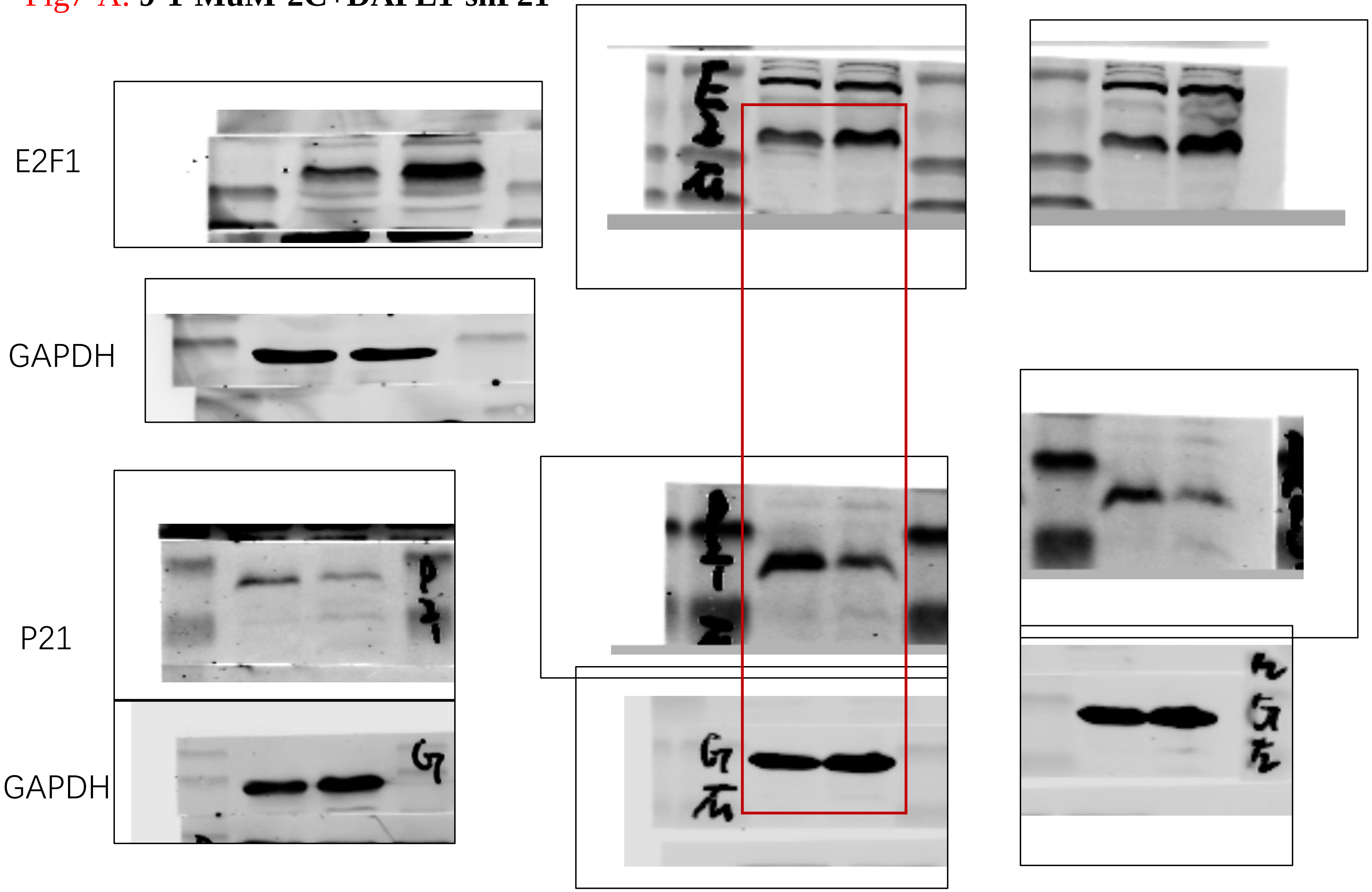

Fig7-A: **9-1-MuM-2C+DAPL1-shP21**

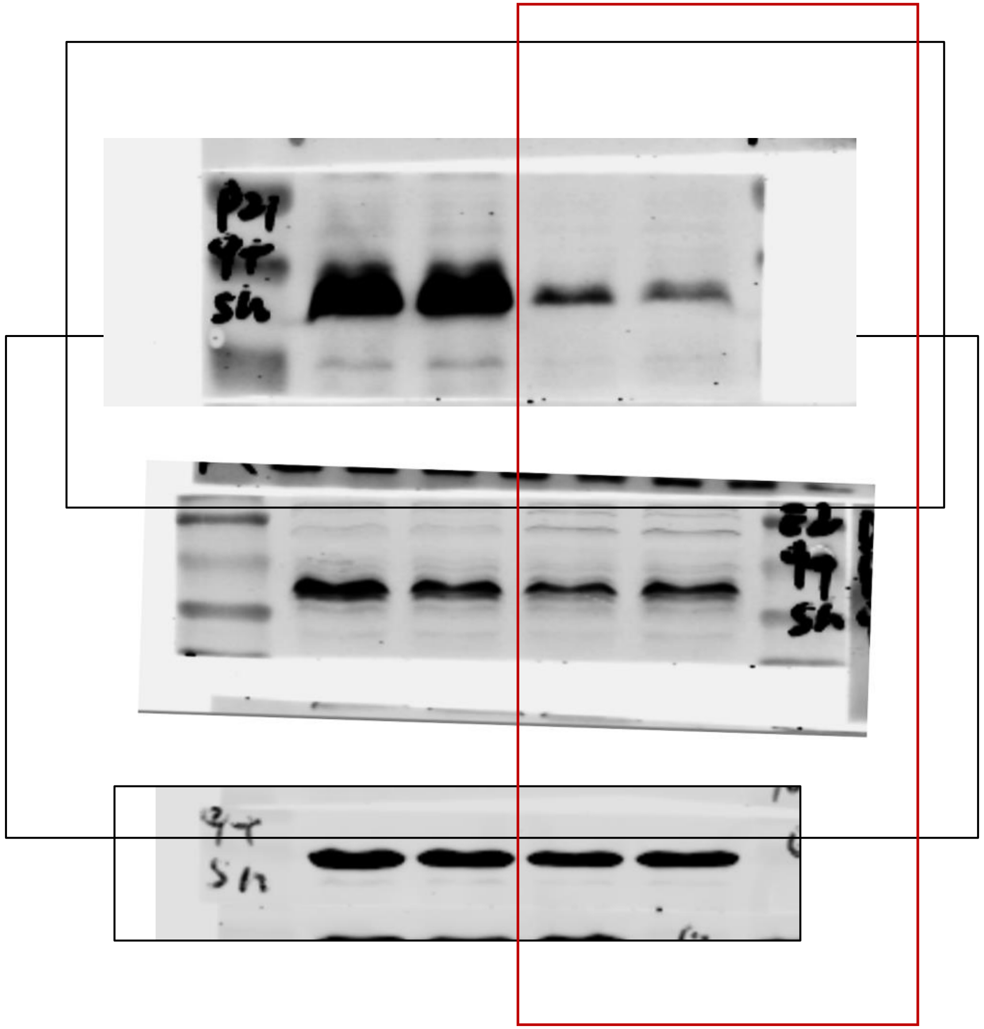

Supplement: Supplementary file 1 — Supplementary file1 (PDF 2465 KB) [file 11010_2024_5067_MOESM1_ESM.pdf]
